# Temozolomide resistance mechanisms: unveiling the role of translesion DNA polymerase kappa in glioblastoma spheroids *in vitro*

**DOI:** 10.1042/BSR20230667

**Published:** 2024-05-29

**Authors:** Diego Luis Ribeiro, Marcela Teatin Latancia, Izadora de Souza, Abu-Bakr Adetayo Ariwoola, Davi Mendes, Clarissa Ribeiro Reily Rocha, André Van Helvoort Lengert, Carlos Frederico Martins Menck

**Affiliations:** 1 Departament of Microbiology, Institute of Biomedical Sciences, University of São Paulo, São Paulo, São Paulo, Brazil; 2 Department of Clinical and Experimental Oncology, Federal University of São Paulo, São Paulo, São Paulo, Brazil; 3 Department of Biophysics, Paulista School of Medicine, Federal University of São Paulo, São Paulo, São Paulo, Brazil

**Keywords:** 3D spheroids, apoptosis, Genotoxicity, Glioma, Temozolomide, Translesion Synthesis

## Abstract

Temozolomide (TMZ) is the leading therapeutic agent for combating Glioblastoma Multiforme (GBM). Nonetheless, the persistence of chemotherapy-resistant GBM cells remains an ongoing challenge, attributed to various factors, including the translesion synthesis (TLS) mechanism. TLS enables tumor cells to endure genomic damage by utilizing specialized DNA polymerases to bypass DNA lesions. Specifically, TLS polymerase Kappa (Polκ) has been implicated in facilitating DNA damage tolerance against TMZ-induced damage, contributing to a worse prognosis in GBM patients. To better understand the roles of Polκ in TMZ resistance, we conducted a comprehensive assessment of the cytotoxic, antiproliferative, antimetastatic, and genotoxic effects of TMZ on GBM (U251MG) wild-type (WTE) and TLS Polκ knockout (KO) cells, cultivated as three-dimensional (3D) tumor spheroids *in vitro*. Initial results revealed that TMZ: (i) induces reductions in GBM spheroid diameter (10–200 µM); (ii) demonstrates significant cytotoxicity (25–200 μM); (iii) exerts antiproliferative effects (≤25 μM) and promotes cell cycle arrest (G2/M phase) in Polκ KO spheroids when compared with WTE counterparts. Furthermore, Polκ KO spheroids exhibit elevated levels of cell death (Caspase 3/7) and display greater genotoxicity (53BP1) than WTE following TMZ exposure. Concerning antimetastatic effects, TMZ impedes invadopodia (3D invasion) more effectively in Polκ KO than in WTE spheroids. Collectively, the results suggest that TLS Polκ plays a vital role in the survival, cell death, genotoxicity, and metastatic potential of GBM spheroids *in vitro* when subjected to TMZ treatment. While the precise mechanisms underpinning this resistance remain elusive, TLS Polκ emerges as a potential therapeutic target for GBM patients.

## Introduction

Glioblastoma multiforme (GBM) is the most aggressive and invasive cancer of the central nervous system (CNS), being designated as ‘the killer’ due to its median survival rate of approximately 14–15 months [1]. Currently, the primary pharmacological intervention for GBM, classified as the first-line treatment, involves the utilization of Temozolomide (3-methyl-4-oxoimidazo [5,1-d][1-3,5] tetrazine-8-carboxamide; TMZ), due to its remarkable clinical efficacy and its unique ability to traverse the blood–brain barrier [2]. TMZ adds a methyl group (-CH_3_) to nitrogenous DNA bases, promoting genotoxic lesions [3]. The genomic lesions promoted by TMZ encompass N7-methylguanine (N7-meG), N3-methyladenine (N3-meA), and O6-methylguanine (O6-meG) [4]. They can generate abasic sites, DNA replication blockage [5], nucleotide substitutions [4], or, in the case of the most genotoxic lesion O6-meG, cause single- and double-strand breaks (SSB/DSBs), cell cycle arrest, and ultimately culminate in cell death [3,6].

Nevertheless, the emergence of chemotherapy-resistant cells to TMZ remains a recurrent and challenging issue in treating GBM patients [7,8]. Several factors contribute to this cell resistance, including genetic and epigenetic alterations, development of cell death resistance mechanisms, and activation of DNA repair pathways, which facilitate the survival of GBM cells [3,9,10]. Furthermore, tumor cells exhibit heightened expression of DNA damage tolerance (DDT) mechanisms, enabling the cells to withstand the genomic lesions induced by chemotherapy agents [11,12]. Within this context, the translesion synthesis (TLS) mechanism emerges as a crucial component of the DDT machinery, triggered when genotoxic lesions obstruct the progress of replicative DNA polymerases. TLS DNA polymerases (TLS pols) come into action to prevent replicative forks’ collapse, thereby ensuring the survival of cancer cells [13,14]. Notably, DNA TLS pols possess an enlarged catalytic site capable of accommodating bulky lesions and replicating genetic material, even when errors may occur, potentially leading to mutations [15].

The Y-family of DNA polymerases is the most well-recognized group within TLS. Within this family, DNA polymerase kappa (Polκ) is a pivotal TLS enzyme known for its remarkable capacity to accommodate and bypass DNA adducts, particularly those resulting from benzo[a]pyrene-guanine (B[a]P-G) induced DNA damage [16]. Polκ’s proficiency extends to effectively bypassing DNA adducts induced by chemotherapeutic agents such as TMZ, mitomycin C (MMC), N-methyl-N-nitrosourea (MNU), and methyl methanesulfonate (MMS), thereby lessening their cytotoxic and mutagenic potential [17]. Besides its well-documented role in mitigating DNA adduct-induced damage, Polκ has emerged as a multifaceted player in DNA repair processes. Notably, it has been observed to (i) be recruited in response to pro-oxidative lesions, (ii) participate in the restoration of replication forks in fragile genomic regions [18], and (iii) contribute to the DNA repair synthesis during nucleotide excision repair (NER) following ultraviolet (UV) radiation [19]. Recent evidence has also suggested a novel Polκ function in activating Chk1 within the DDR pathway [20].

Specifically, TLS Polκ exhibits a grade-dependent upregulation in GBM patients. This phenomenon correlates with TMZ chemoresistance, with Polκ established as a well-known prognostic marker predictive of unfavorable clinical outcomes [21,22]. Intriguingly, in recurrent GBM tumors, there is an even more pronounced enrichment of TLS Polκ, highlighting the essential role of this polymerase in tumor cell survival. Conversely, targeting TLS pols presents a promising avenue for sensitizing neoplastic cells to DNA-damaging agents, potentially disrupting their ability to withstand genotoxic stress [14]. Notably, findings from our research group have shed light on the involvement of TLS Polκ in mediating TMZ resistance in glioma cells (U138) *in vitro* [23]. Furthermore, by generating CRISPR-Cas9 mutated GBM cells with ablated TLS Polκ, our group revealed diminished cell viability, heightened genotoxic stress, and cell cycle arrest following TMZ treatments [24]. These observations emphasize the significance of TLS Polκ’s roles in driving GBM chemoresistance and offer promising prospects for developing clinical strategies to improve treatment outcomes.

Nonetheless, more robust models are imperative to provide more pertinent insights into the roles played by TLS pols in TMZ chemoresistance. Within this context, three-dimensional (3D) cell culture models *in vitro* offer a valuable approach, providing a significant representation of the intricate relationship between solid tumors and their microenvironment, surpassing the limitations of conventional two-dimensional (2D) monolayer cultures [25,26]. Among the various 3D models, multicellular tumor spheroids are the most well-characterized and widely accepted option. These spheroids faithfully recapitulate the tumor microenvironment, resembling avascular tumor nodules and closely mirroring *in vivo* conditions. This fidelity is achieved by recreating essential elements such as the extracellular matrix (ECM), intercellular interactions, authentic pathophysiological conditions, and functional tissue properties akin to *in vivo* systems [27]. These features make 3D tumor spheroids the standard model in GBM research to study tumor growth, metastasis and to investigate mechanisms underlying the response to anticancer drugs [27].

The primary objective of the present study is to comprehensively explore the potential roles played by TLS Polκ in mediating resistance to the chemotherapy drug TMZ in GBM U251MG cells cultured as 3D tumor spheroids *in vitro*. As part of our strategy, we evaluated the responses of U251MG cells with TLS Polκ CRISPR-engineered knockout (KO). Our study seeks to unravel the involvement of TLS Polκ by examining its impact on a spectrum of cellular responses, including antiproliferative, cytotoxic, genotoxic, antimetastatic, and cell death responses in a 3D cell culture model. This comprehensive analysis aims to shed light on the underlying mechanisms that govern TMZ chemoresistance in the challenging context of GBM.

## Material and methods

### Temozolomide (TMZ)

Temozolomide (TMZ; ≥ 98% HPLC; C_6_H_6_N_6_O_2_; CAS: 85622-93-1; PM: 194.15 g/L) was obtained from Sigma-Aldrich (St. Louis, MO, U.S.A.) and fully diluted in solution stock (50 mM) in dimethyl sulfoxide (DMSO; CAS 67-68-5; Sigma-Aldrich), being kept at −80°C, in the absence of light. Working solutions (5–200 µM) were diluted from the stock to carry out the biological assays. The TMZ concentrations were based on Rosso et al. [28]. All working solutions were prepared 10× concentrated using culture medium, whereas 3D culture treatments were performed by replacing 10% of the total volume. The aliquots used to treat 3D tumor spheroids were diluted to obtain final concentrations of a maximum of 2% DMSO in the culture medium. To avoid exceeding the DMSO limit, 50% of the culture medium in each well was renewed/replaced every 48 h.

### Cell lines, culture conditions, and gene editing

The U251MG cell line, derived from human glioblastoma astrocytoma, was kindly provided by Dr Bernd Kaina of the University of Mainz (Germany). U251MG TLS Pol Kappa (Polκ) mutated cell line was established using CRISPR/Cas9 gene editing by Latancia M.T. et al. [24]. All production procedures (lentiviral particles, transfection, transduction, selection of clones, and knockout KO validation) are described in Supplementary Data I in Figure S1. U251MG cells containing the empty vector (without sgRNA or ‘empty vector’) were also established as an experimental control model by the same procedure as described for Polκ KO cells and were named U251MG Wild-Type Empty Vector (WTE). The U251MG WTE and TLS Polκ KO cells were cultured in DMEM High Glucose medium (HG; Gibco, Carlsbad, CA, U.S.A.) with 10% Fetal Bovine Serum (FBS; Gibco), 1% antibiotic/antimycotic mix (10000 units/mL penicillin, 10000 µg /ml streptomycin and 25 µg/ml amphotericin B; Gibco) and 0.024% sodium bicarbonate (Sigma-Aldrich). In culture, U251MG cells were maintained in T25 and T75 cm^2^ bottles (Nunc™ Cell Culture Flasks; ThermoFisher Scientific) in a 3110 Series II CO_2_ Water Jacketed incubator (ThermoFisher Scientific) with an atmosphere of 5% of CO_2_ at 37°C and relative humidity of 96% until reaching 70–80% of confluence. Subsequently, the cells were detached using TrypLE™ Express enzyme (1X; Gibco) and maintained under the described conditions. The procedures for maintaining cell lines followed the recommendations proposed by Bal-Price and Coecke [29]. For 3D spheroids experiments, only cell aliquots between the third and eigth passage were used. The U251MG cells authentications (including TLS Polκ KO) were performed by the Multiuser Equipment Network Program (PREMiUM) at the Hospital das Clínicas da Faculdade de Medicina da USP (HC/FMUSP) using STR DNA typing (Short Tandem Repeat) according to the International Reference Standard for Authentication of Human Cell Lineages with a panel of 8 STR loci (D5S818, D13S317, D7S820, D16S539, vWA, TH01, TPOX, and CSF1P0) plus gender determination (AMEL). All U251MG cells were regularly tested for mycoplasma contamination by MycoAlert® PLUS Mycoplasma Detection Kit (Cat. Nº LT07-710; Lonza, Basel, Switzerland).

### 3D tumor spheroids culture (Agarose Coated-Overlay)

The 3D tumor spheroids from U251MG WTE and TLS Polκ KO cells were cultured according to Friedrich et al. [30] by the agarose-coated overlay methodology. Adaptations using methylcellulose (MC; Cat. Nº M0512; Sigma-Aldrich) were based on Longati et al. [31] and Guyon et al. [32]. Previously, a 12% MC stock solution was prepared, and the working solution was diluted at 2% MC solution using DMEM HG. For generation of 3D tumor spheroids, U251MG WTE and TLS Polκ KO (1.0 × 10^4^) cells were seeded into each well of a 96-well plate (Nunc™; ThermoFisher Scientific) previously coated with agarose (Invitrogen) at 2.0% (m/v) in 1× PBS (pH 7.4). For this, the agarose solution was autoclaved for 20 min at 121°C and, after autoclaving, the flask containing the hot solution was transferred to laminar flow, after that 75 μl were pipetted into a well with the Multipette pipette plus 10 (Eppendorf; Munich, Germany). After solidification, complete DMEM HG medium containing U251MG cells was mixed with 2% MC solution (0.20% final), and 100 μl/well was dispensed using an 8-channel manual micropipette (HTL Discovery Corning; Warszawa, Poland). Finally, the plates were centrifuged at 350 × ***g*** for 10 min, then transferred to an incubator with 5% CO_2_ at 37°C and 96% humidity and immobile for 96 h. After initiation, the U251MG GBM 3D spheroids were checked for uniformity, completeness, and 300–500 µm diameter (with proliferative, quiescent, and necrotic zones). After validation, 3D spheroid cultures were used for TMZ treatments.

### Experimental design

The treatment protocol with TMZ in 3D tumor spheroids of U251MG WTE cells and TLS Polκ KO was based on the multimodal treatment performed with GBM patients undergoing chemotherapy [33]. After radiotherapy, GBM patients undergo chemotherapy with TMZ in cycles, each lasting 1–5 days (usually 5) with 2-day intervals of up to 28 days. Therefore, an initial screening with various concentrations of TMZ (up to 200 µM) was performed with 1 (120 h/Day 5) and 2 cycles (240 h/Day 10) in the initial screening using cell viability (resazurin) and antiproliferative (integrity/diameter) parameters. Then, four concentrations of TMZ (25, 50, 100, and 200 µM) were selected to analyze cytotoxicity, survival, cell cycle, cell death, migration/invasion, and genotoxicity with times of 24, 48, 72, 96, and 120 h. Solvent Control (SC; DMSO 1%) and Positive Control (PC; Cisplatin 100 µM; Cat Nº P4394; Sigma-Aldrich).

### Cell viability

#### Resazurin assay

Cell viability assessment using the resazurin reduction assay (AlamarBlue®; CAS: 62758-13-8; Sigma-Aldrich) was performed according to Riss et al. [34] at Day 5 (120 h) and Day 10 (240 h) of TMZ treatment. Briefly, after treatments with Negative Control (NC; DMEM HG), SC (DMSO 1%), PC (Cisplatin; 100 µM), and TMZ (5–200 µM), a resazurin working solution (0.15 mg/ ml) was diluted in PBS (pH 7.4) and, of this solution, 40 µl (20% final volume) were added to each well containing the GBM spheroids. The plates were then incubated together at the last 24 h in an oven at 37°C, and then the fluorescence was measured in a Glomax Discover Microplate Reader System (Promega; Madison, WI, U.S.A.) with excitation/emission λ = 530–560/590 nm. Finally, the fluorescence was converted into a percentage (%) of cell viability by normalizing the fluorescence of each treatment with the fluorescence of the NC, which is considered 100% cell viability.

### CellTiter-Glo 3D ATP assay

The cell viability assay based on intracellular ATP quantification was performed by luminescence with the CellTiter-Glo® 3D Reagent (Cat. Nº G9681; Promega) following the manufacturer’s instructions. Briefly, after treatments with SC (DMSO 1%), PC (Cisplatin; 100 µM), and TMZ (25–200 µM) at Day 5 (120 h) and Day 10 (240 h), the U251MG WTE and TLS Polκ KO spheroids (in 50 µl of culture medium) were transferred using a Pasteur pipette to white 96-well plates with clear bottoms (Nunc MicroWell 96-Well Optical-Bottom Plates; Cat. Nº 165306) and 50 µl of CellTiter-Glo® 3D Reagent was then added to each well. After that, the plate was taken to the Glomax Discover Microplate Reader System (Promega), where it underwent a lysis step (shaking orbit = 2 mm and 300 cycles/min) for 5 min, a 25-min luminescent signal stabilization step (room temperature) and, finally, the luminescence reading (RFU) was performed. The luminescence (RFU) obtained was transformed into cell viability (% intracellular ATP), followed by normalization of the luminescence of each treatment with those of the SC, considered 100% cell viability.

### Diameter, morphology, and integrity analyses

Diameter, morphology, and integrity analyses of 3D U251MG WTE from cells and TLS Polκ KO spheroids were performed following Friedrich et al. [30] and Vinci et al. [35]. After initiation, photomicrographs of the 3D tumor spheroids were obtained after Day 0, Day 5 (120 h), Day 7 (168 h), and Day 10 (240 h) treatment with SC (1% DMSO), PC (Cisplatin 100 µM) and TMZ (5–200 µM). All images were taken with the EVOS™ XL Core Imaging System (Thermo Fisher Scientific) capture system, using a 10× objective. The analyses of the photomicrographs were carried out using the Zen 3.1 software. Blue Edition (Carl Zeiss; Berlin, Germany). The integrity/morphology analysis evaluated each image to detect irregular spheroids (without a circular shape) with disaggregation or irregular cell agglomeration. For diameter analysis, the circumference of the 3D spheroid was measured using Zen 3.1 software using the ‘Measure’ tool. The diameter comprises presented in μm.

### Cell survival

#### Clonogenicity

The 3D clonogenic assay was performed based on Mikhail et al. [36]. Briefly, 3D spheroids from U251MG WTE cells and TLS Polκ KO were treated until Day 5 (120 h) with SC (1% DMSO), PC (Cisplatin 100 µM), and TMZ (25–200 µM). After the treatments, the 3D spheroids were collected and transferred to a 1.5 ml microtube (Eppendorf) and centrifuged for 5 min at 300 ×***g*** at room temperature. A sample consisted of six spheroids (*n*=6) to obtain the required number of cells. Once centrifuged, the supernatant was removed, and TrypLE™ enzyme was added to dissociate the 3D spheroids. The tubes were kept in an oven at 37°C for 5–10 min, being manually shaken every 1–2 min. After dissociation, a complete medium was added and homogenized, and each tube was centrifuged again for 5 min at 300 × ***g***. Then, the supernatant was discarded, the resulting pellet was resuspended, and the cell count was performed with Trypan blue (0.4%) in a Countess™ Automated Cell Counter (Thermo Fischer Scientific). In total, 3,000 cells from the pellet were taken and seeded in 6-well plates (Greiner Bio-One) with complete medium. After 10 days, the colonies were fixed for 10 min with 10% Formaldehyde and stained with 0.4% Crystal Violet (Sigma-Aldrich) for 10 min. Finally, the colonies were manually counted. The survival fraction (SF) was calculated according to Franken et al. [37].

### Genotoxicity

#### 53BP1trunc-apple reporter plasmid

For genotoxicity analysis, U251MG WTE and TLS Polκ KO cells containing the 53BP1 reporter plasmid fused with the Apple fluorescent protein [38] were established to determine the DNA double-strand breaks (DSBs) accumulation. Initially, 53BP1trunc-Apple plasmid (Addgene; Cat. Nº 27698) was amplified using *Escherichia coli* bacteria grown in LB medium with ampicillin, followed by plasmid DNA extraction using a MidiPrep kit (Qiagen). The lentiviral particles were produced in HEK-293FT cells by co-transfecting the plasmid with the helper plasmids, psPAX2, and pMD2, using polyethyleneimine (PEI). After 48 h, the supernatant medium was collected, filtered (0.45 μm; cellulose acetate), and viral particles were concentrated by ultracentrifugation. The transduction of lentiviral particles in U251MG cells was performed in 6-well plates (0.5 × 10^6^) for 24 h. After that, the medium was replaced and maintained for another 48 h. Following this period, individualized U251MG cells were grown to form clones. After 15 days, colonies were collected and transferred to 96-well plates and, after reaching adequate confluence, expanded until freezing. The U251MG cells with regular 53BP1trunc-Apple expression were confirmed by fluorescence microscopy. The 3D spheroids of U251MG 53BP1trunc-Apple cells were initiated in 96-well black plates with U-shaped bottom clear (CellStar® Chimney µClear®; Greiner Bio-One) with 2.0% agarose in PBS, being transferred to a CO_2_ incubator at 37°C and 96% humidity for 96 h. After initiation, treatments with TMZ (25–100 µM), SC (1% DMSO), and PC (Cisplatin 100 µM) were performed and photomicrographs acquired at times of 0-, 24-, 48-, and 72-h using EVOS XL Core Imaging System with the RFP channel (Apple) and the plates were scanned using the ‘one field of view per well’ acquisition mode with the 10× objective. The images were analyzed using Fiji v.3.1 [39] using the ‘automatic particle counting’ after background adjustment by the ‘image threshold’. The minimum and maximum size (2–100 µm) was also adjusted to exclude anything other than cells within the 3D spheroid; scale bar: 400 µm.

### Cell cycle

#### Fast-FUCCI reporter plasmid

For cell cycle analysis, were established U251MG WTE and Polκ KO cells containing the reporter plasmid pBOB-EF1-FastFUCCI-Puro (Addgene; Cat. Nº 86849). This plasmid has the expression of CDT1 and its inhibitor, Geminin [40]. In eukaryotic cells, the peak of CDT1 levels occurs during G1 and decreases upon entry into the S phase. Conversely, the geminin levels are higher during S/G2 but fall during mitosis and G1 phase [41]. By fusing the fluorescent proteins Kusabira Orange 2 (mKO; red) with CDT1 and Azami Green (mAG) with geminin, the FastFUCCI system allows the visualization of living cells in the G1 (red) or S (yellowish) or S/G2-M phases (green). The processes of plasmid amplification, production of lentiviral particles, transfection, transduction, and selection were the same as described in the genotoxicity section. Furthermore, U251MG spheroids expressing FastFUCCI were also initiated as described for 53BP1 and, after initiation, treated with TMZ (25–100 µM), SC (1% DMSO), and PC (cisplatin: 100 µM). The photomicrographs of 3D tumor spheroids were acquired on 0, Day 3, and Day 5 using the EVOS XL Core Imaging System image capture system with the channels FITC (mAG) and RFP (mKO) merged. The plates were scanned using the ‘one field of view per well’ acquisition mode with the 10× objective. The acquired images were analyzed using Fiji v.3.1 [39] using ‘color histogram counting’. The presence of cells in the G1/S (red; RFP), S (yellow), and G2/M (green; FITC) phases were quantified by the ‘average fluorescence intensity’ on each channel. Representative images from U251MG spheroids expressing the FastFUCCI plasmid are shown with Red/RFP and Green/FITC merged; scale bar: 400 µm.

### Cell death

#### Apoptosis (Caspase Activity 3/7)

The apoptotic cell death analysis in 3D spheroids of U251MG WTE and TLS Polκ KO cells was performed with the Caspase-Glo® 3/7 kit (Cat. Nº G8091; Promega) following the manufacturer’s instructions after treatments with SC (DMSO 1%), PC (cisplatin: 100 µM) and TMZ (25–200 µM) by Day 5 (120 h). Briefly, the 3D tumor spheroids in culture medium (50 µl) were transferred using a Pasteur pipette to white 96-well white plates with a transparent bottom (Thermo Scientific™ Nunc MicroWell 96-Well Optical-Bottom Plates, Cat. Nº 165306) and 50 µL of Caspase-Glo® 3/7 Reagent was added to each well. After that, the plate was taken to the Glomax Discover Microplate Reader System (Promega), where it underwent a reaction/stabilization step of the luminescent signal for 35 min at room temperature. Finally, the luminescence reading (RFU) was performed, which was transformed in Caspase 3/7 activity (%) by normalizing the luminescence of each treatment with SC.

#### Necrosis (propidium iodide staining)

Necrotic cells were analyzed using Propidium Iodide (Thermo Fischer Scientific, Cat. Nº P3566) dye. Briefly, after initiation, U251MG WTE and TLS Polκ KO spheroids were treated with SC (1% DMSO), PC (cisplatin: 100 µM), and TMZ (25–200 µM). Along with the treatments, PI (10 μg/ml in PBS) and Hoechst 33342 (10 μM in PBS; Thermo Fischer Scientific; Cat. Nº H3570) were added to each well, and the samples were analyzed at 0, Day 3, and Day 5 (120 h). The acquisition parameters were RFP (Channel 1; PI), and DAPI (Channel 2; Hoechst 33342) merged. The ‘autofocus’ was set to ‘brightfield’, and the plates were scanned with acquisition mode ‘one field of view per well’ in the 10× objective. The images were obtained by fluorescence microscopy using the EVOS XL Core Imaging System image capture system. The acquired images were analyzed using Fiji v.3.1 [39] using ‘color histogram counting’. Necrotic cells were quantified using the ‘average fluorescence intensity’ recorded on the RFP channel. Representative images from U251MG spheroids are shown with Hoechst (Blue/DAPI), and PI (Red/RFP) merged; scale bar: 400 µm.

### Metastasis

#### 3D cell migration assay (ECM Gel)

The analysis of 3D cell migration from U251MG WTE and TLS Polκ KO spheroids to the Extracellular Matrix (ECM) was performed with Matrigel® Matrix (Phenol Red-free, LDEV-free; Cat Nº 356237; Corning) based on Vinci et al. [42]. Initially, the Matrigel solution (10 mg/ml) was diluted in incomplete DMEM HG medium (200 μg/ml final concentration) and then pipetted into each well of the 96-well plate (Nunc™ MicroWell™; Cat. Nº 167008; ThermoFisher Scientific), remaining at room temperature for 3 h to fix the ECM to the well bottom. Subsequently, the remaining non-adherent volume was carefully removed, the wells were washed with PBS, and a 1% Bovine Albumin Serum (BSA; Sigma-Aldrich; Cat. Nº A2153) in PBS 1× solution was added for 1h. Then, the initiated 3D spheroids, together with complete DMEM HG medium, were transferred to plates ECM coated and then supplemented with medium containing treatments with SC (DMSO 1%), PC (Cisplatin; 100 µM), and TMZ (25–200 µM). After 30 min, images of each well corresponding to time 0 (*t* = 0) were acquired with the EVOS™ XL Core Imaging System capture system, using a 10× objective. The remaining images were obtained after 24 and 48 h. For the analysis, each image was evaluated by measuring the circumference of the migrated cells around the spheroid with the help of the Zen 3.1 software (Carl Zeiss) with the ‘Measure’ tool. The diameters are presented in μm.

#### 3D tumor invasion assay

The analysis of 3D invasion with U251MG WTE and TLS Polκ KO spheroids was performed according to Vinci et al. [43] and Berens et al. [44]. Briefly, after 3D tumor spheroids initiation using 96-well plates U-bottom (Cat. Nº 3358; Corning), 150 μl/well of culture medium was removed from each well, and the dishes (containing the 3D spheroids) kept on ice for 30 min. During this time, an adhesion medium with Matrigel (Phenol Red-free, LDEV-free; Cat. Nº 356237; Corning) was prepared on the ice (4 mg/ml final concentration). Carefully, after homogenizing, 100 μl of the Matrigel adhesion medium was added, the bubbles were removed, and the plates were transferred to a CO_2_ incubator at 37°C for 1 h to ECM solidification. Then, 50 μl of DMEM HG complete culture medium was added with the addition of treatments with SC (DMSO 1%), PC (Cisplatin 100 µM), and TMZ (25–200 µM) above the solidified adhesion medium. In the end, a photomicrograph of each 3D spheroid was recorded at 0, 24, and 48 h using the EVOS™ XL Core Imaging System microscope at the 10× objective. After capturing the images, the diameter covered by the 3D spheroids was measured using the Zen 3.1 Blue Edition software (Zeiss) using the ‘Measure’ tool in μm. The change in diameter (invaded core x spheroid core) at each time point was calculated concerning at time = 0 h. Finally, the data were converted into cell invasion percentages (% t0).

### Statistical analysis

The results obtained were initially submitted to analyze the distribution normality by Shapiro–Wilk test. As the results showed a parametric distribution, the data were analyzed using ANOVA (analysis of variance) one-way (comparisons with SC) followed by Dunnett’s post-test and ANOVA two-way (comparisons with WTE cells) followed by Bonferroni post-test, considering *P*≤0.05 as significant. All analyses were performed using GraphPad Prism 9.0 software (La Jolla, CA, U.S.A.).

## Results

### TMZ-induced reduction in the diameter of 3D GBM Polκ KO spheroids

**Figure 1:**
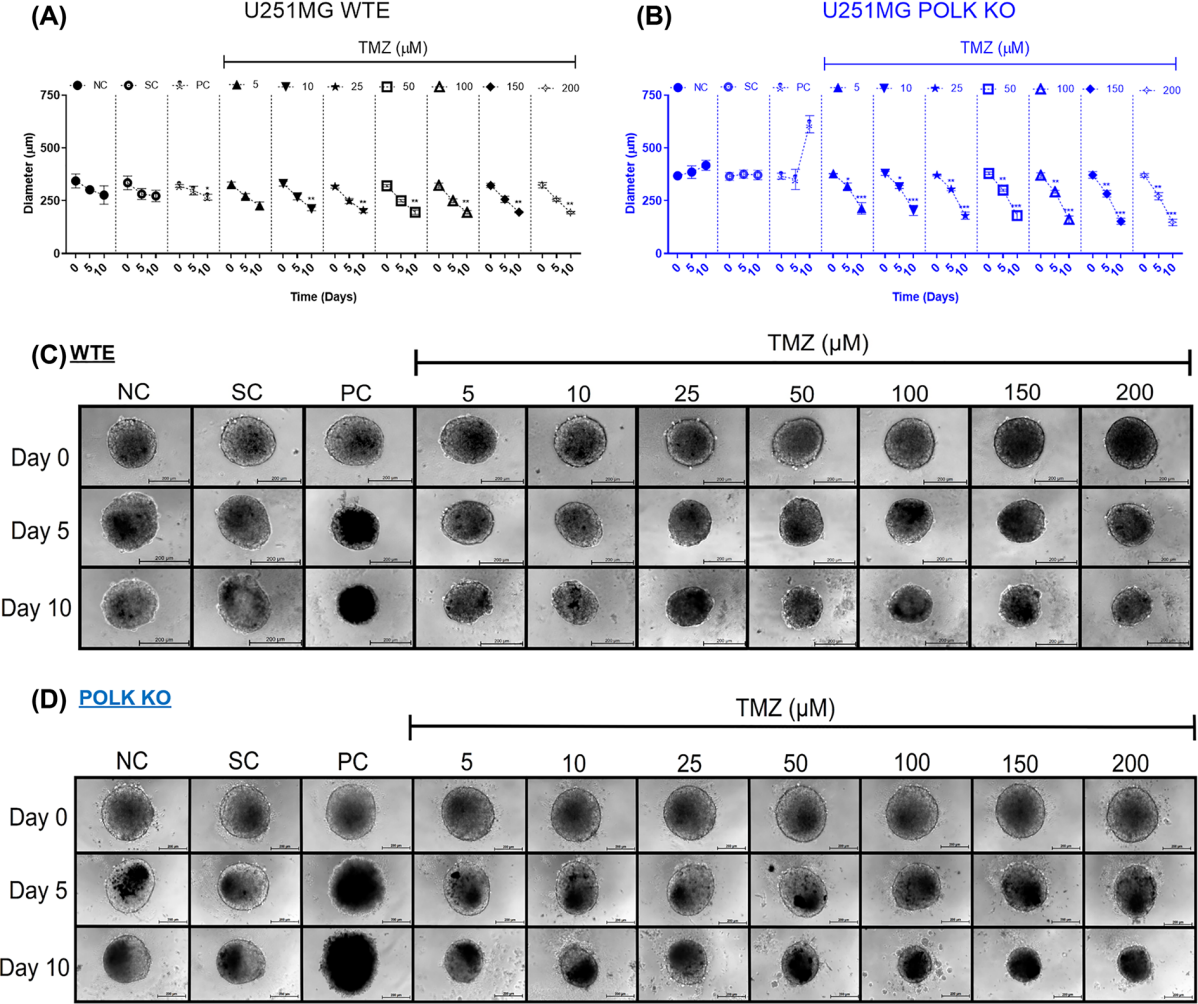
Diameter, morphology, and integrity of GBM spheroids after TMZ treatment Diameter (µm) of U251MG WTE (**A**) and TLS Polκ KO (**B**) 3D tumor spheroids treated with TMZ and respective controls at Day 0, Day 5, and Day 10. Morphology and Integrity of U251MG WTE (**C**) and TLS Polκ KO (**D**) spheroids treated with TMZ and respective controls at Day 0 and Day 10. All images were obtained using the EVOS XL microscope, using the 10x objective. All data were represented as means ± standard deviation (*X* ± SD) from six spheroids (*n*=6) per replicate and three independent biological experiments (*n*=3). *Values statistically different from the NC group at the point (date) (*P<0.05, **P<0.01, ***P<0.001; ANOVA followed by Dunnett’s post-test). NC: Negative Control (DMEM HG); PC: Positive Control (Cisplatin 100 μM); SC: Solvent Control (1% DMSO); TMZ: Temozolomide. Scale: 200 μm.

The results of diameter, morphology, and integrity analyses conducted in 3D tumor spheroids of U251MG WTE and TLS Polκ KO cells following TMZ treatment and respective controls are shown in Figure 1. Initially, TMZ (ranging from 5 to 200 µM) led to notable decreases in the WTE spheroids diameter, statistically significant only after Day 10 compared with the negative control (NC, only medium) (Figure 1A,C). In contrast, in U251MG Polκ KO spheroids, all TMZ concentrations exhibited a significant decrease in spheroid diameter on Day 5 (Figure 1B) across all TMZ concentrations tested (5 to 200 µM), compared with NC and SC (1% DMSO). On Day 10, TMZ induced even more substantial reductions in the diameter of Polκ KO spheroids (Figure 1B,D). The SC (1% DMSO) showed no difference from the NC (DMEM HG). Curiously, 3D GBM spheroids treated with cisplatin (PC, positive control) substantially increased in diameter on Day 10. Possibly, this effect is related to the breakdown in the central region that promoted disaggregation and diameter increase.

### GBM Polκ KO spheroids show enhanced sensitivity to TMZ-induced cytotoxicity

**Figure 2:**
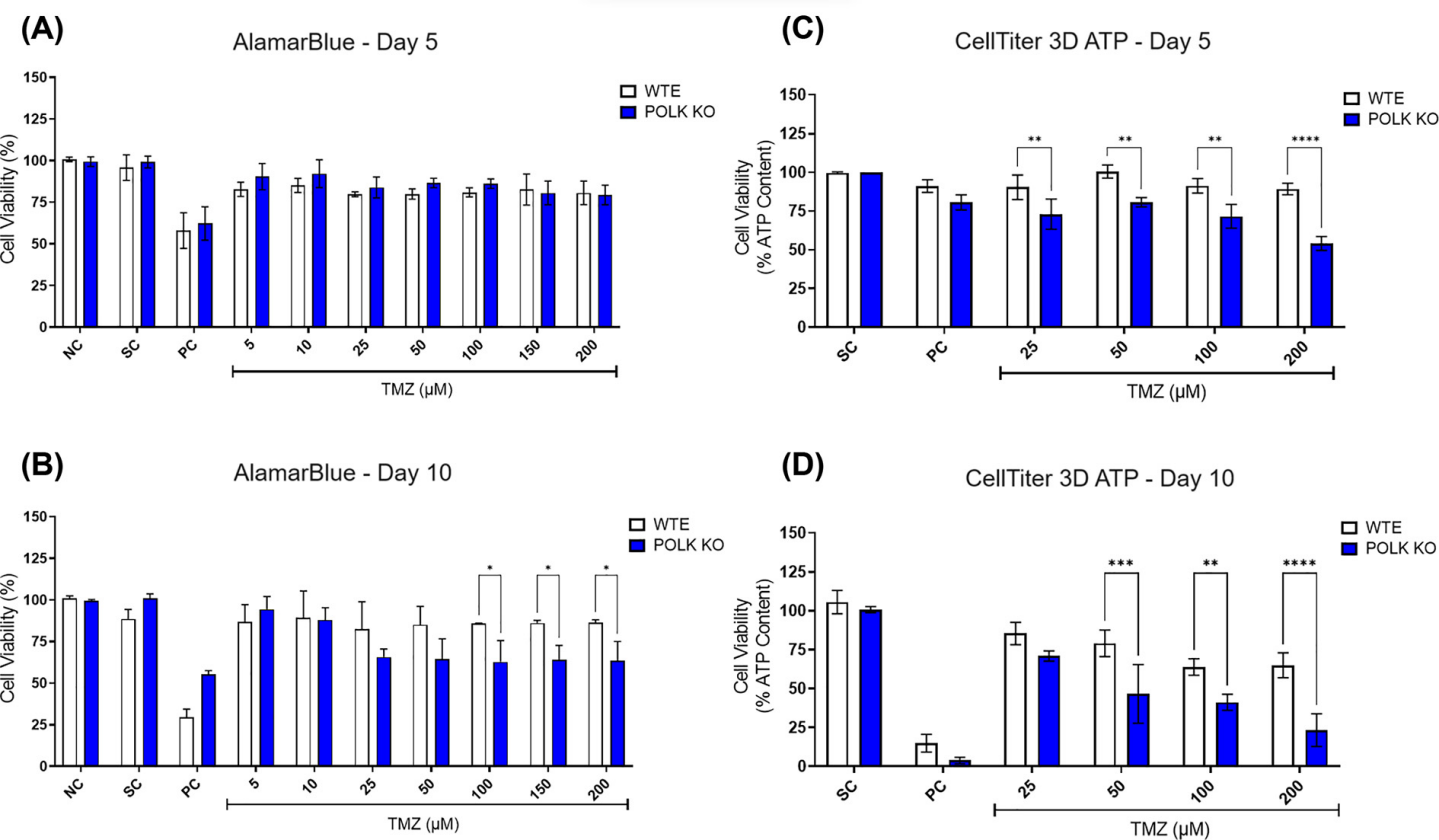
Cell viability assessment in GBM spheroids following TMZ treatment (**A,B**) Cell viability (%) of U251MG WTE and TLS Polκ KO 3D tumor spheroids after exposure with TMZ and their respective controls at Day 5 (**A**) and Day 10 (**B**) as assessed by the resazurin assay. (**C,D**) Cell viability (% ATP Content) of U251MG WTE and TLS Polκ KO tumor spheroids after treatments with TMZ and respective controls at Day 5 (**C**) and Day 10 (**D**) as evaluated by CellTiter-Glo 3D ATP cell viability assay. All data were represented as means ± standard deviation (*X* ± SD) from four spheroids (*n*=4) per replicate to resazurin and three spheroids (*n*=3) per replicate to CellTiter-Glo ATP and three independent biological experiments (*n*=3). *Values statistically different from the WTE cells at the point (date) (*P<0.05, **P<0.01, ***P<0.001, ****P<0.0001; Two-way ANOVA followed by Bonferroni post-test). NC: Negative control (DMEM HG); PC: Positive Control (Cisplatin 100 μM); SC: Solvent Control (1% DMSO); TMZ: Temozolomide.

The results of the resazurin cell viability assay conducted on 3D GBM spheroids following TMZ treatment are depicted in Figure 2 and Supplementary Figure S2. In U251MG WTE spheroids, TMZ (5–200 µM) did not elicit a reduction in viability on either Day 5 or Day 10. Conversely, in TLS Polκ KO spheroids, TMZ concentrations of ≥25 μM decreased cell viability from Day 5, with significant differences observed compared with WTE spheroids on Day 10. Subsequently, the assays were carried out at 25, 50, 100, and 200 µM concentrations. Further evaluation using CellTiter 3D ATP assay confirmed a reduction in percentage (%) ATP content in U251MG TLS Polκ KO spheroids across the tested TMZ concentrations on Day 5 and Day 10. In contrast, GBM WTE spheroids showed no significant impact on viability on Day 5 and Day 10. Upon direct comparison, it becomes evident that TMZ substantially reduces cell viability in Polκ KO spheroids, particularly on Day 10.

### TMZ impairs cell survival, particularly in GBM TLS Polκ KO spheroids

**Figure 3:**
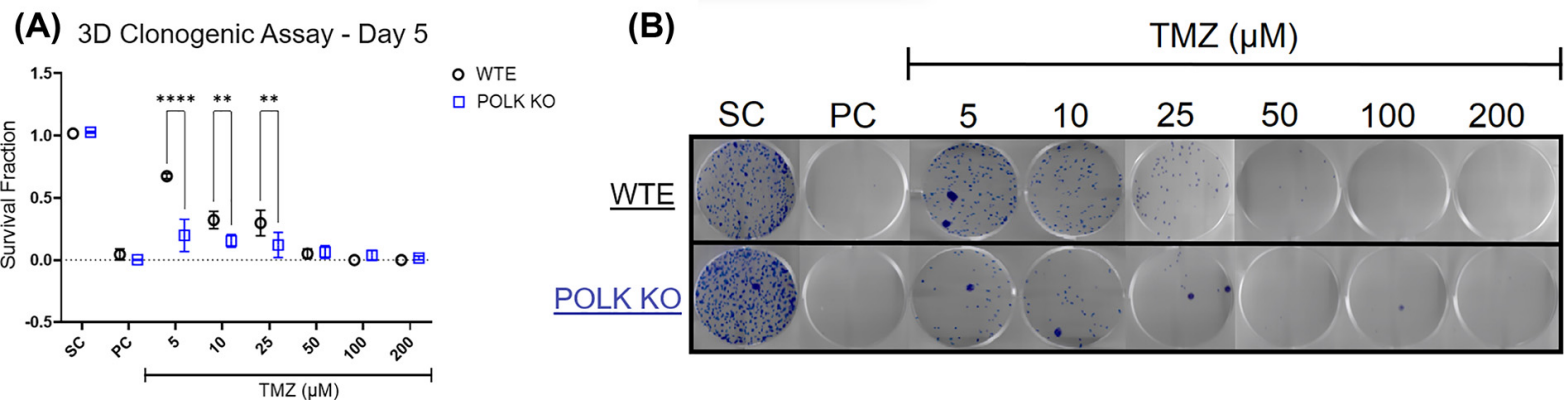
Cell survival in GBM spheroids after TMZ treatment (**A**) Survival Fraction (SF) of U251MG WTE and TLS Polκ KO cells disaggregated from 3D tumor spheroids treated with TMZ and respective controls until Day 5. In the assay, colonies formed after 10 days of 3000 cells disaggregated from 3D tumor spheroids were analyzed. (**B**) Photomicrograph of the colonies formed after 10 days of disaggregated cells from 3D tumor spheroids. All data were represented as means ± standard deviation (*X* ± SD) from six spheroids (*n*=6) per well and three independent biological experiments (*n*=3). *Values statistically different from the U251MG WTE cells at the point (date) (**P<0.01, ***P<0.001, ****P<0.0001; Two-way ANOVA followed by Bonferroni post-test). PC: Positive Control (Cisplatin 100 μM); SC: Solvent Control (1% DMSO); TMZ: Temozolomide.

The cell survival was assessed in U251MG WTE and TLS Polκ KO spheroids following TMZ treatment, and results are shown in Figure 3 and Supplementary Figure S3. The results revealed that all TMZ concentrations reduced survival fraction (SF) after disaggregation of 3D GBM spheroids treated up to Day 5. Interestingly, even at the lowest TMZ concentrations (5, 10, and 25 μM), the TLS Polκ KO GBM spheroids significantly reduced survival compared to WTE GBM cells. Remarkably, at TMZ concentrations of ≥50 μM, clones were very low for WTE cells and TLS Polκ KO so that no significant differences could be detected. Particularly noteworthy are the outcomes observed with the lowest TMZ concentration (5 μM), where the antiproliferative effects in U251MG TLS Polκ KO cells exhibited the highest significance (*P*<0.0001).

### TLS Polκ KO spheroids show earlier cell cycle changes after TMZ treatments

**Figure 4:**
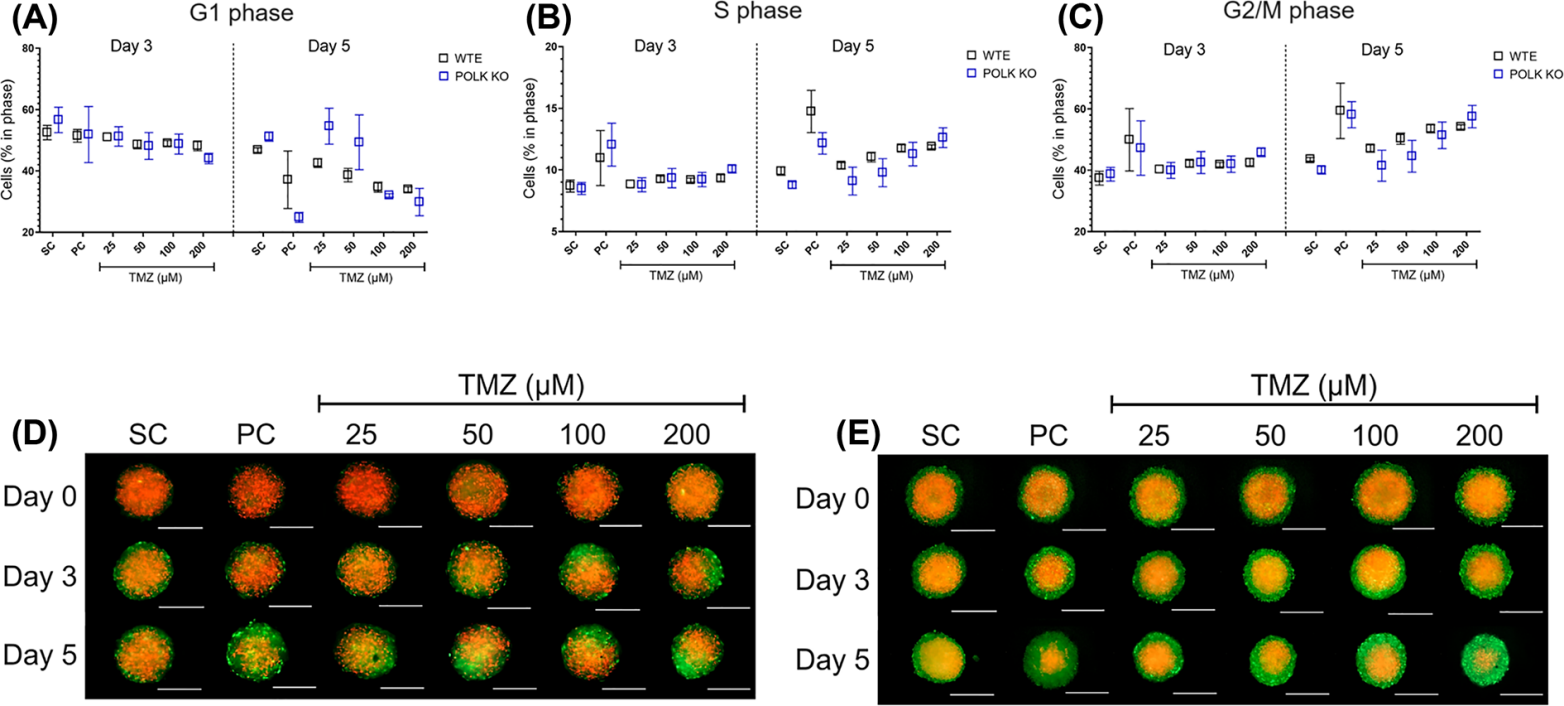
Analysis of cell cycle in GBM spheroids following TMZ treatment (**A,B**) Cell Population (%) in each cell cycle phase in U251MG WTE (**A**) and TLS Polκ KO (**B**) 3D tumor spheroids expressing pBOB-EF1-FastFUCCI-Puro plasmid after Day 0, Day 3, and Day 5 of treatments with TMZ and their respective controls. (**C,D**) Photomicrographs of 3D tumor spheroids of U251MG WTE (**C**) and TLS Polκ KO (**D**) tumor spheroids after Day 0, Day 3, and Day 5 of treatment with TMZ and respective controls. All images were obtained by the EVOS XL core microscope using an RFP (red) and FITC (green) channel and using a 10× objective. The acquired images were analyzed and quantified by the software Fiji v. 3.1. Scale: 400 μm (white bar). All data were represented as means ± standard deviation (*X* ± SD) from four spheroids (*n*=4) per replicate and three independent biological experiments (*n*=3). No values statistically different from the WTE cells at the point (date) (Two-way ANOVA followed by Bonferroni post-test). PC: Positive Control (Cisplatin 100 μM); SC: Solvent Control (1% DMSO); TMZ: Temozolomide.

The results with GBM 3D tumor spheroids expressing the FastFUCCI plasmid following TMZ treatment and respective controls at Days 0, 3, and 5 are presented in Figure 4 and Supplementary Figure S4. After pairwise comparisons, no statistical differences were observed at each cell cycle phase between WTE and TLS Polκ KO GBM spheroids. At Day 3, TMZ ≥ 50 µM increases cell population in S and G2/M phase and decreases G1 cells in TLS Polκ KO spheroids. Interestingly, after cisplatin (PC; 100 μM) treatment, the TLS Polκ KO spheroids show a clear green color, indicating most cells are blocked in G2/M. At the same time (Day 3), no alterations in cell cycle phases were detected in WTE spheroids. By Day 5, we still observe a substantial number of cells that seem to be arrested in G2/M (green) after treatments with TMZ (≥50 μM) and cisplatin (PC) in TLS Polκ KO GBM spheroids. In WTE GBM spheroids, despite increasing G2/M, the presence of cells in G1 after treatments with cisplatin and TMZ (≥100 μM) is observed in 3D GBM spheroids.

### TLS Polκ KO spheroids exhibit enhanced genotoxicity levels following TMZ treatment

**Figure 5:**
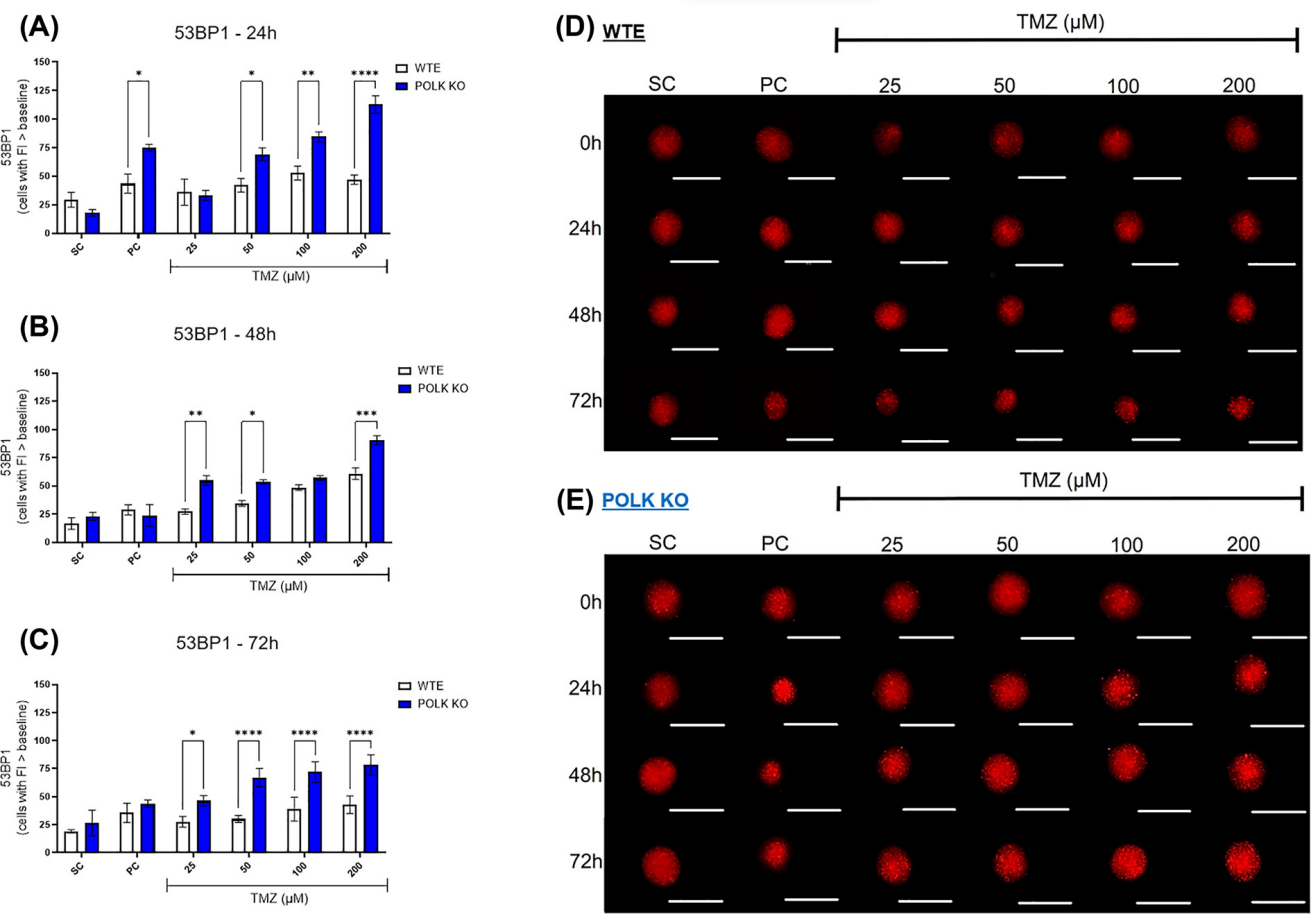
Evaluation of genotoxicity in GBM spheroids following TMZ treatment (**A–C**) Comparison of the number of cells with 53BP1-Apple-trunc fluorescence above the baseline in U251MG WTE and TLS Polκ KO 3D tumor spheroids evaluated after 24- (A), 48- (B), and 72-h (C) treatments with TMZ and their respective controls. (**D,E**) Photomicrographs of 3D tumor spheroids of U251MG WTE (D) and TLS Polκ KO (E) cells obtained after treatment 0, 24, 48, and 72 h with TMZ and respective controls. All images were obtained by the EVOS XL core microscope using an RFP (red) channel and 10× objective. The images were analyzed and quantified by the software Fiji v. 3.1. Scale: 400 μm (white bar). All data were represented as means ± standard deviation (*X* ± SD) from four spheroids (*n*=4) per replicate and three independent biological experiments (*n*=3). *Values statistically different from the WTE cells at the point (date) (*P<0.05, **P<0.01, ***P<0.001; Two-way ANOVA followed by Bonferroni post-test). PC: Positive Control (Cisplatin 100 μM); SC: Solvent Control (1% DMSO); TMZ: Temozolomide.

The results with 3D GBM tumor spheroids expressing the 53BP1 trunc-Apple following TMZ treatment and respective controls are presented in Figure 5 and Supplementary Figure S5. After 24 h, TMZ concentrations of ≥50 µM initially increased 53BP1 fluorescence in TLS Polκ KO spheroids compared with the SC group. Subsequently, after 48 h, TMZ concentrations of ≥100 μM significantly increased 53BP1 levels in WTE spheroids. By 72 h, TLS Polκ KO displayed statistically significant increases in 53BP1 fluorescence at TMZ concentrations of ≥50 μM. When the response to TMZ is compared between WTE and TLS Polκ KO spheroids, statistical analysis demonstrates the sensitivity of Polκ KO spheroids to genotoxic effects induced by TMZ from ≥50 μM after 24, 48, and 72 h.

### GBM Polκ KO spheroids exhibit a higher increase in cell death parameters

**Figure 6:**
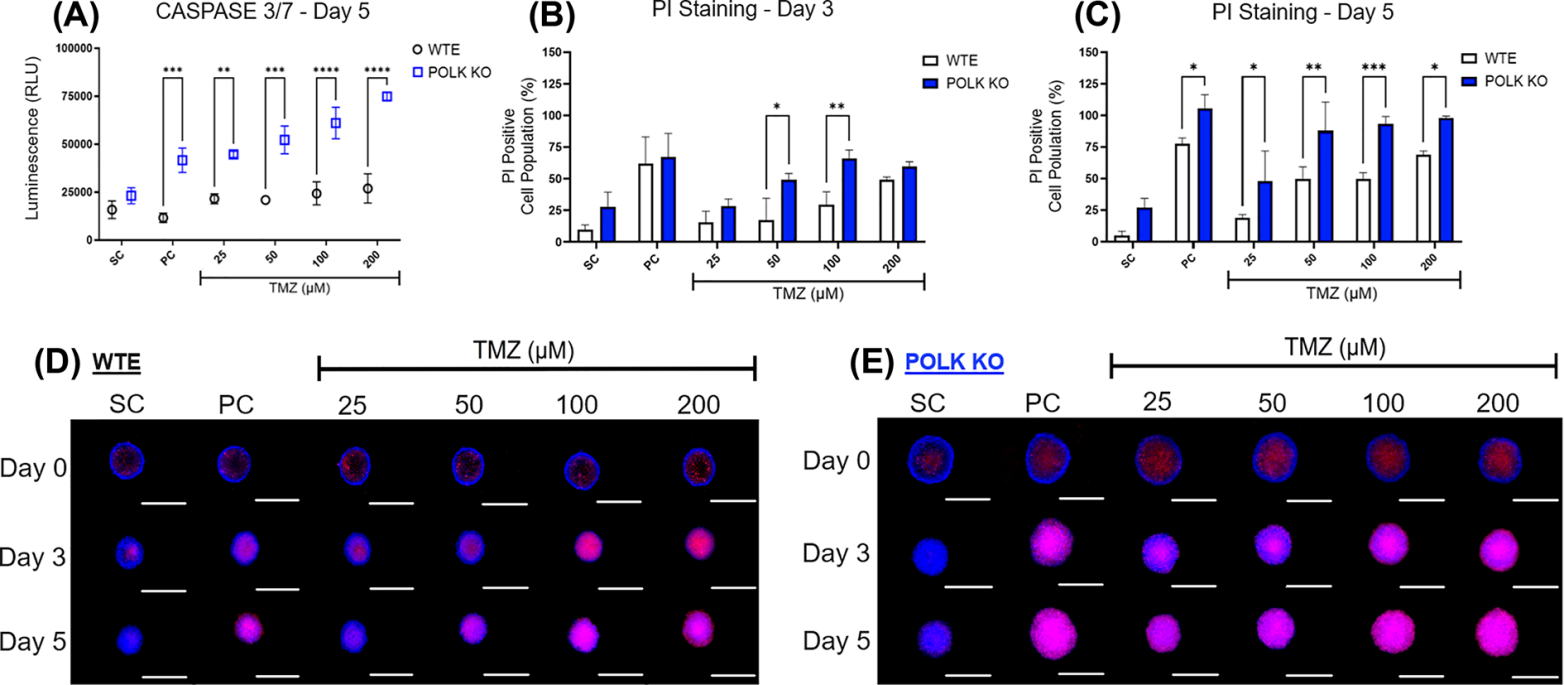
Assessment of cell death parameters in GBM spheroids following TMZ treatment (**A**) Relative Luminescence (RLU) of Caspase 3/7 activity in U251MG WTE and TLS Polκ KO 3D tumor spheroids after treatments with TMZ and respective controls at Day 5. (**B,C**) Cell Population (%) with Positive Propidium Iodide (PI) staining fluorescence in 3D tumor spheroids from U251MG WTE (B) and TLS Polκ KO (C) cells after treatments with TMZ and respective controls at Day 3 and Day 5. (**D,E**) Photomicrographs of U251MG WTE (D) and TLS Polκ KO (E) tumor spheroids obtained after treatments with TMZ and respective controls at Day 0, Day 3, and Day 5. All images were obtained by the EVOS XL core microscope using RFP (PI) and DAPI (Hoechst) channels and with the 10× objective. The acquired images were analyzed and quantified by the software Fiji v. 3.1. Scale: 400 μm (white bar). All data were represented as means ± standard deviation (*X* ± SD) from four spheroids (*n*=4) per replicate and three independent biological experiments (*n*=3). *Values statistically different from the U251MG WTE cells at the point (date) (*P<0.05, **P<0.01, ***P0.001;,****P<0.0001; Two-way ANOVA followed by Bonferroni post-test). PC: Positive Control (Cisplatin 100 μM); SC: Solvent Control (1% DMSO); TMZ: Temozolomide.

To quantify the extent of cell death induced by TMZ in GBM spheroids, we conducted the apoptosis/necrosis assays, and the results are shown in Figure 6 and Supplementary Figure S6. Initially, we measured Caspase 3/7 levels, a marker of apoptosis induction, and the quantification reveals that TMZ ≥ 100 μM increased Caspases 3/7 levels in Polκ KO spheroids compared with the SC group (Supplementary Figure S6). In WTE spheroids, TMZ increased Caspase 3/7 levels only at 200 μM concerning the SC group. Upon comparison, TLS Polκ KO spheroids displayed increased Caspase 3/7 levels at all TMZ concentrations compared with WTE. Next, we subjected GBM 3D spheroids treated with TMZ to PI staining. The quantification revealed that TMZ ≥ 100 μM increased the population of PI-positive cells on Day 3 in Polκ KO spheroids. In WTE spheroids, an increase of PI-positive cells was observed only at the highest TMZ concentration (200 μM). By Day 5, TMZ ≥ 50 μM significantly increased the number of PI-positive cells in WTE and Polκ KO 3D spheroids compared with SC. Upon side-by-side analysis in each treatment group, the population of PI-positive cells observed in TLS Polκ KO spheroids was consistently higher across all TMZ concentrations. A PI experiment was also performed to check for necrosis in another TLS Polκ KO clone. Both clones were sensitive to TMZ, as shown in Supplementary Figure S6.

### TMZ inhibits more effectively tumor invasiveness in TLS Polκ KO spheroids

**Figure 7:**
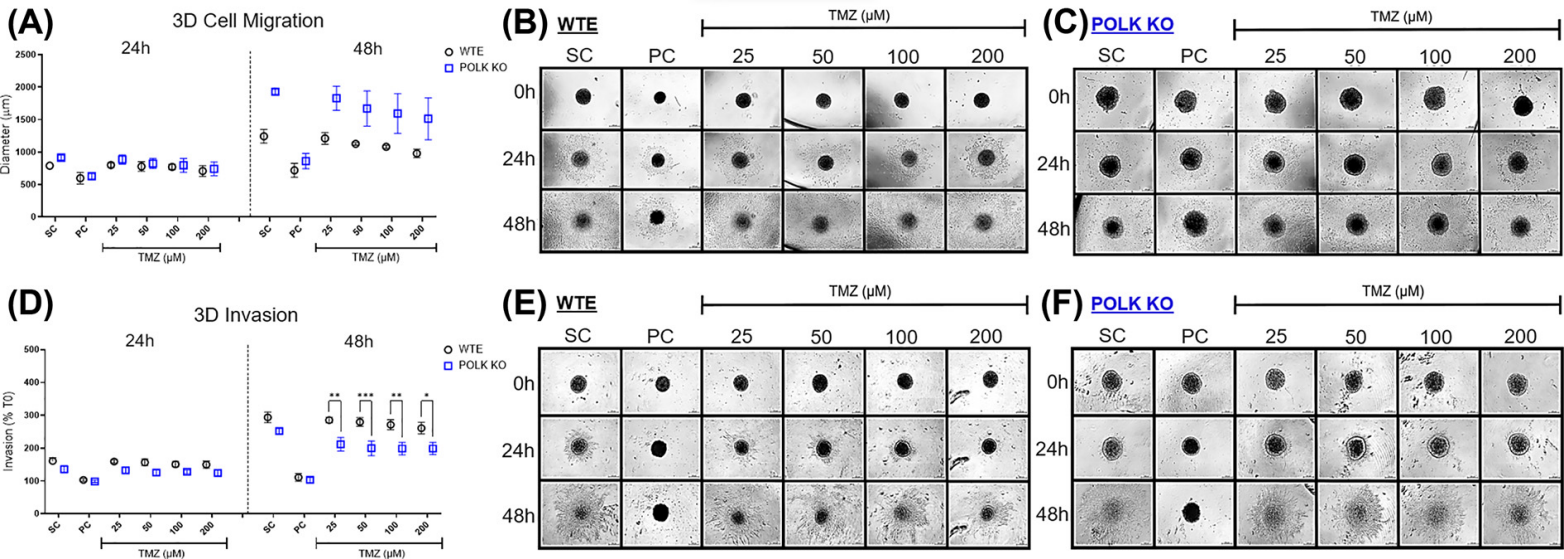
Quantifying metastatic parameters in GBM spheroids following TMZ treatment (**A**) Comparison of diameter increase (µm) covered by U251MG WTE and TLS Polκ KO 3D tumor spheroids after treatments with TMZ and respective controls for 0, 24, and 48 h. The diameter (µm) of migration of the 3D spheroids to the ECM after 24 and 48 h were converted into % to the increase in diameter obtained at 0 h. (**B,C**) Photomicrographs of U251MG WTE (B) and TLS Polκ KO (C) 3D tumor spheroids in Matrigel® obtained after 0-, 24-, and 48 h of treatment with different TMZ concentrations and their respective controls. The photomicrographs were obtained by the EVOS™ XL microscope using the 10x objective. (**D**) Comparison of percentage (%) of cell invasion (invadopodia) formed by U251MG WTE and TLS Polκ KO 3D tumor spheroids after treatments with TMZ and its respective controls for 0-, 24- and 48 h. The invasion area (µm^3^) into the ECM (Matrigel) was converted to % considering the diameter increase of 0 h. (**E,F**) Photomicrographs of U251MG WTE (E) and TLS Polκ KO (F) 3D tumor spheroids in Matrigel were obtained after 0-, 24-, and 48 h of treatment with TMZ and respective controls. The photomicrographs were obtained by the EVOS™ XL microscope using the 10× objective. The diameter analyses were performed using Zen 3.1 software (Carl Zeiss). Scale bar: 200 μm. All data were represented as means ± standard deviation (*X* ± SD) from four spheroids (*n*=4) per replicate and three independent biological experiments (*n*=3). *Values statistically different from the U251MG WTE cells at the point (date) (*P<0.05, **P<0.01, ***P<0.001, ****P<0.0001; Two-way ANOVA followed by Bonferroni post-test). PC: Positive Control (cisplatin: 100 μM); SC: Solvent Control (1% DMSO); TMZ: Temozolomide.

The 3D cell migration/invasion in U251MG WTE and TLS Polκ KO spheroids treated with TMZ and respective controls are depicted in Figure 7 and Supplementary Figure S7. After 24 h, only TMZ 200 µM decreased migration in Polκ KO spheroids. After 48 h, TMZ ≥ 50 μM reduced migration in Polκ KO, while in WTE spheroids, these effects were achieved only for TMZ concentrations ≥ 100 μM. Notably, when the results were compared, there were no discernible differences in 3D migration between WTE and TLS Polκ KO spheroids. Curiously, considering controls (SC), Polκ KO spheroids displayed an unexpectedly increased diameter, not observed in WTE spheroids. The 3D invasion assay revealed that TMZ concentrations of ≥100 μM suppressed invadopodia formation in Polκ KO spheroids after 48 h. In contrast, WTE spheroids displayed no significant response to TMZ treatment. Comparative analyses unequivocally demonstrated that TMZ reduced 3D invasion capacity in TLS Polκ KO cells compared with the WTE counterparts.

## Discussion

GBM stands as the most prevalent neoplasm affecting the central nervous system. It is characterized as highly aggressive, with considerable tumor heterogeneity, discouraging prognosis, and slow and poor response to treatment. Currently, the recommended therapeutic approach for GBM patients involves surgery for tumor resection (when feasible), radiotherapy, and adjuvant chemotherapy [45]. The most common drug in chemotherapy is TMZ, a well-absorbed oral medication with the critical ability to traverse the blood–brain barrier (BBB), a crucial aspect in treating GBM patients. However, a substantial number of GBM relapses post-TMZ chemotherapy can be attributed to the activation of DDT mechanisms, given the need to overcome threats to genome replication [46].

One of these DDT mechanisms is TLS, where specific DNA polymerases play a pivotal role. The Y-family polymerases (Polη, Polκ, Polι, and Rev1) have emerged as prominent subjects to promote the bypass of genomic lesions induced by chemotherapy drugs (including TMZ). Their activity may increase the tumors cells’ capacity to overcome the replicative stress, facilitating continued cell proliferation [47]. Furthermore, TLS processes may contribute to tumor heterogeneity, as these polymerases are error-prone and can generate mutations and increase basal mutagenesis [48]. Therefore, the Y-Family TLS pols play a multifaceted role in influencing disease progression, patient responses to therapy, and tumor recurrence [49]. At the same time, they have become essential in understanding the intricacies of tumor biology and are currently one of the newest and most promising fronts in therapeutic strategies [50].

Research studies have already showcased the promising potential of inhibiting TLS pols in sensitizing tumor cells to chemotherapeutic agents. The inhibition of TLS Polι sensitizes lung cancer cells to cisplatin treatment [51]. Jansen et al. revealed that inhibiting TLS pol Rev1 sensitized chronic myeloid leukemia (CML) cells to genotoxic chemotherapeutic agents, increasing DNA damage and downregulating stem cell-related genes [52]. Srivastava et al. reported the potential of a small-molecule inhibitor (Chrysin) that targets TLS Polƞ in sensitizing ovarian cancer stem-like cells (CSLCs) to cisplatin treatment [53]. Moreover, TLS Polκ depletion (using siRNA) on DRG neurons treated with cisplatin reveals a significant reduction in DNA repair mechanisms and higher DNA damage levels [18].

Specifically, concerning TLS pol’s role in mediating the resistance to TMZ chemotherapy in GBM cells, our research group has previously listed TLS Polι and Polκ as potentially associated with TMZ resistance for GBM (U138MG) cells [23]. Latancia et al. [10] have shown that human XPV cells, which lack Polη, exhibited more sensitivity to TMZ treatment than proficient cells. This sensitivity was accompanied by increased cell cycle arrest (G2/M phase) and elevated levels of genotoxicity, as indicated by increased levels of γ-H2AX [10]. Moreover, recent data showed that GBM U251MG TLS Polκ and Polι KO (CRISPR-Cas9) cells displayed reduced cell viability and induced cell cycle arrest following TMZ treatment [24]. However, despite these compelling findings, there was a need to analyze the effects of TMZ using more robust *in vitro* culture models and explore the potential of TLS pols inhibition in the context of GBM chemotherapy.

The 3D tumor spheroids have proven to be considerably more resistant to chemotherapy drugs than cancer cells cultured in 2D monolayers. The enhanced resistance is attributed to their structural organization, multi-layer cellular arrangement, hypoxic regions, and nutrient gradients, all of which closely resemble the microenvironment of human tumors [32]. GBM spheroids are acknowledged for their ability to fine-emulate tumor microenvironment conditions and replicate the biological responses observed in GBM chemoresistance [54]. In the present study, employing 3D tumor spheroids, we observed that TMZ led to significant cell viability reduction, mainly in U251MG Polκ KO spheroids. Additionally, our findings underscore the differences in biological responses between 2D and 3D culture models. In WTE spheroids, even the highest TMZ concentration (200 μM) failed to reduce cell viability by 50%. In 2D monolayers, the IC50 values after the same 5-day TMZ treatment regimen typically hover approximately 50–60 µM [55]. These differences align with previous estimates for 3D cultures, where IC50 values can exhibit increases from 5 to 50 times in comparison with results obtained from 2D cultures [56–58].

Following cell viability assays, we evaluated the diameter, morphology, and integrity of the 3D spheroids [59]. The diameter and integrity assessment holds particular significance in appraising the effectiveness of cancer therapies, as it mirrors the effects observed in *in vivo* tumors [60]. Besides, we also evaluated cell survival, which was employed to characterize better the antiproliferative effects of TMZ on 3D GBM spheroids. The 3D clonogenic assay, which involves the disaggregation of cells from GBM spheroids, provides a valuable means to discern quiescent and senescent tumor cells that have challenged the chemotherapy treatment within the 3D spheroid and subsequently resumed their proliferation [61,62]. The tumor quiescence/senescence can arise after chemotherapy, constituting one of the fundamental underlying mechanisms of TMZ resistance in GBM [63]. The results showed that TMZ promoted a 3D spheroid diameter reduction and decreased cell survival in GBM Polκ KO cells.

Similar results were observed in cell cycle analysis using the reporter plasmid pBOB-EF1-FastFUCCI-Puro. By Day 5, TMZ ≥ 50 μM induced cell cycle arrest in S and G2/M and decreased G1 population in GBM Polκ KO spheroids. Then, WTE spheroids also demonstrated similar results, although less striking, on Day 5. Previous data showed cell cycle arrest in the G2/M phase after treatments with TMZ at 72 and 120 h in GBM lines cultured at 2D monolayers [64,65]. The results obtained with the FastFUCCI system using living 3D GBM spheroids are the first to indicate sensitivity on the cell cycle promoted by TMZ chemotherapy. In essence, our results demonstrate that TLS Polκ KO cells within 3D spheroid structures may experience challenges in resisting TMZ and, as a result, exhibit a heightened propensity to undergo a cell death process.

To discern the responses to cell death between GBM spheroids, we assessed parameters related to apoptosis/necrosis, once that the best-known mechanism of resistance to TMZ by GBM cells is the inhibition of cell death [66]. In the Caspase 3/7 assay, TLS Polκ KO spheroids exhibited higher levels at Day 5 treatment when exposed to TMZ. These results corroborate previous studies demonstrating TLS Polκ associated with GBM tumors by apoptosis resistance following TMZ treatments [21,67]. The ‘effector’ caspases 3 and 7 are activated in the apoptosis cascade, resulting in proteolytic degradation [68]. Likewise, we observed an increase in the population of PI-positive cells after TMZ treatments in Polκ KO compared to WTE spheroids. PI is a red-fluorescent DNA intercalating dye that can only permeate the membrane of non-viable cells, indicating that necrosis or late apoptosis arises from homeostasis dysregulation, where proteases degrade cell components, and the nuclear membrane becomes disrupted [69].

Notably, the primary factor associated with cell death activation in GBM cells is TMZ-induced DNA damage. To assess whether the genotoxicity contributes to TMZ sensitivity, DNA damage induction was measured using the fluorescent reporter plasmid 53BP1trunc-apple, where the truncated version of the 53BP1 protein binds to DNA double-strand breaks (DSBs) [38]. The GBM Polκ KO spheroids exhibited elevated 53BP1 fluorescence levels when treated with TMZ (≥50 μM) for 24 and 72 h. Ketkar et al. [70] also reported increased DNA damage (alkaline comet assay) in HAP-1 cells Polκ deficient (CRISPR/Cas9) treated with TMZ (100 μM) or in HAP-1 proficient cells treated with TMZ but in combination with IAG-10, a specific pharmacological TLS Polκ inhibitor. The collective data strongly suggest that GBM Polκ KO cells have increased genomic instability after TMZ treatment. Notably, this study was the first to measure 53BP1 levels in 3D GBM spheroids using cells transfected with a reporter plasmid.

Currently, cells that manage to escape from the primary tumor and subsequently give rise to metastases are recognized as the principal culprits behind chemotherapy resistance. Therefore, beyond antiproliferative and cytotoxic effects, antitumor therapies should understand mechanisms capable of retarding the formation of metastases [71]. Cellular migration/invasion plays a pivotal role in metastases, where tumor cells move away from the original tumor site to colonize secondary areas. Such movement may result in the infiltration or colonization of surrounding tissue sites [42]. Moreover, tumor cells can disseminate to distant organs by forming specialized structures known as ‘invadopodia’. These structures are actin filaments and proteinases, responsible for motility, ECM degradation, and leakage into vascular channels, facilitating hematogenous (or lymphatic) dissemination and allowing the tumor cells to travel to remote organ sites [43,72].

To evaluate metastatic parameters, the 3D migration and the 3D invasion assays were performed with the U251MG WTE and TLS Polκ KO spheroids on plates coated with Matrigel, an ECM solution extracted from animal tumors that contain laminin, type IV collagen, heparin sulfate proteoglycan, entactin and other secondary components of solid tumors. The 3D invasion assay revealed that TLS Polκ KO spheroids significantly reduced invadopod formation compared with WTE following TMZ treatment. It is worth noting that our data obtained with GBM spheroids represent pioneer contributions in investigations of metastatic parameters. The effects of TMZ on cell invasion are likely associated with genomic instability and cell death perceived in similar conditions.

Following a comprehensive analysis of all results on integrity/diameter, viability, cell death, genotoxicity, and 3D migration/invasion, pronounced differences in responses to TMZ were detected between WTE and TLS Polκ KO spheroids. GBM Polκ KO spheroids exhibited enhanced sensitivity to TMZ treatment at lower concentrations and treatment periods across most evaluated parameters. TLS Polκ, in addition to its role in bypassing DNA adducts, may activate ATR-Chk1 kinases in the DNA damage response (DDR) pathway, thereby contributing to the resolution of replicative stress through other repair pathways, such as Homologous Recombination (HR) Repair [20,70]. The low expression/recruitment of TLS Polκ to DNA replication forks can increase genomic instability even without genotoxic treatment [73]. Outwardly, once Polκ is dysregulated, a diminishment of DDR capacity is observed and could contribute to DSB formation.

Conversely, the overexpression or enrichment of TLS Polκ has been linked to tumor resistance to TMZ chemotherapy in GBM patients, often implying a poor prognosis [21]. Therefore, due to its ability to circumvent DNA damage and its role in DDR (and resolution of replicative stress), targeting TLS Polκ either pharmacologically or genetically may provide an alternative to GBM patients undergoing TMZ chemotherapy [74,75]. In fact, Ketkar et al. [70] demonstrated that IAG-10, a pharmacological TLS Polκ inhibitor, can potentiate the antiproliferative and genotoxic activity of TMZ in human HAP-1 cells *in vitro*.

In conclusion, our findings underscore the promising therapeutic potential association with the inhibition of TLS Polκ in the chemotherapy regimen of GBM patients. These strategies of TLS Polκ inhibition can be achieved either genetically (e.g., using small interference RNA or CRISPR KO) or pharmacologically by developing specific chemical inhibitors. Moreover, this work demonstrates the instrumental role played by 3D tumor spheroids as they recapitulate the GBM tumor microenvironment, contributing to a more precise representation of responses to TMZ treatment. Therefore, this work not only affirms that 3D GBM spheroids TLS Polκ KO are more sensitive to the antiproliferative, cytotoxic, genotoxic, and antimetastatic effects of TMZ but also underscores the promising therapeutic potential of TLS Polκ inhibition in the chemotherapy of GBM patients.

## Supplementary Material

Supplementary Figures S1-S7 and Supplementary Data I

## Data Availability

All data that support the findings of this study are available from the corresponding author upon reasonable request.

## Abbreviations

53BP1: P53 binding protein 1
ANOVA: analysis of variance
ATP: adenosine triphosphate
BBB: blood–brain barrier
BSA: Bovine Serum Albumin
CRISPR: Clustered Regularly Interspaced Short Palindromic Repeats
DAPI: 4′,6-diamidino-2-phenylindole
DDR: DNA damage response
DDT: DNA damage tolerance
DMEM HG: Dulbecco's Modified Eagle Medium High Glucose
DMSO: dimethyl sulfoxide
DSB: double-strand break
ECM: extracellular matrix
FITC: fluorescein channel
GBM: glioblastoma multiforme
H2Ax: histone H2AX
HR: homologous recombination
IAG-10: indole-aminoguanidine 10
IC50: half maximal inhibitory concentration
KO: knockout
NC: negative control
NIH: National Institutes of Health
PBS: phosphate-buffered saline
PC: positive control
PI: propidium iodide
Polη: translesion DNA polymerase eta
Polι: translesion polymerase iota
RFP: red fluorescent protein
RFU: relative fluorescence units
SC: solvent control
SF: survival fraction
sgRNA: single-guide RNA
SSB: single- strand break
TLS Polκ: translesion DNA polymerase Kappa
TLS: translesion synthesis
TMZ: temozolomide
WTE: wild-type empty-vector
XPV: Xeroderma pigmentosum variant
