# Supplementary material for: Temozolomide resistance mechanisms: unveiling the role of translesion DNA polymerase kappa in glioblastoma spheroids in vitro
Source: Biosci Rep. 2024 May 29;44(5):BSR20230667. doi: 10.1042/BSR20230667 (PMC11139666; doi:10.1042/BSR20230667)
Supplement: Supplementary Figures S1-S7 and Supplementary Data I [file BSR-2023-0667C_supp.pdf]

Supplementary Information

Temozolomide Resistance Mechanisms: Unveiling the role of Translesion DNA Polymerase Kappa in Glioblastoma Spheroids *in vitro*

Diego Luis Ribeiro[1]*, Marcela Teatin Latancia[1, #], Izadora de Souza[2], Abu-Bakr Adetayo Ariwoola[1,2], Davi Mendes[1], Clarissa Ribeiro Reily Rocha[2], André Van Helvoort Lengert[3], and Carlos Frederico Martins Menck[1†]

[1] Departament of Microbiology, Institute of Biomedical Sciences, University of São Paulo, São Paulo, São Paulo, Brazil.

[2] Department of Clinical and Experimental Oncology, Federal University of São Paulo, São Paulo, São Paulo, Brazil.

[3] Department of Biophysics, Paulista School of Medicine, Federal University of São Paulo, São Paulo, São Paulo, Brazil.

[#]Present Address: Laboratory of Genomic Integrity, National Institute of Child Health and Human Development, National Institutes of Health, Bethesda, MD, USA.

*Correspondence:

Carlos Frederico Martins Menck, Ph.D.

DNA Repair Laboratory
Department of Microbiology - Institute of Biomedical Sciences (ICB) – University of São Paulo (USP). Avenida Prof. Lineu Prestes, Nº 1374. Sala 116, CEVAT Gene II. Ed. Biomédicas 2.
São Paulo, São Paulo, Brazil.
ZipCode: 05508-000
Phone : +55.11.3091.7499
E-mail : cfmmenck@usp.br

Supplementary Data I

Material and Methods

Gene Editing (CRISPR/Cas9)

U251MG TLS Pol Kappa (Polκ) mutated cell line was established by CRISPR/Cas9 by Latancia M.T. *(submitted).* Briefly, the lentiCRISPRv2 plasmids (AddGene; Cat. Nº #52961) containing the specific guide RNA (sgRNA) were designed with 20 base pairs (bp) complementary to the region of interest in the genome. The sgRNA sequence was immediately followed by the PAM sequence, which, in the case of the Cas9 enzyme, is 5'-NGG-3'. The sgRNA was annealed and cloned into a lentiCRISPRv2 plasmid under the control of the U6 promoter, which was amplified using *Escherichia coli*. The bacteria carrying the plasmid were cultured in an LB medium with ampicillin, followed by plasmid DNA extraction. Lentiviruses were produced by co-transfecting the plasmid lentiCRISPRv2 cloned with sgRNA for Polκ with plasmids pMD2.G (Addgene; Cat. Nº #12259) and psPAX2 (Addgene; Cat. Nº #12260) into HEK-293FT cells (human embryonic kidney cells). After 72 h, the supernatant with the lentiviruses was collected, filtered, and concentrated by ultracentrifugation. Viruses were resuspended in minimal DMEM medium, aliquoted into cryovials, and frozen at -80°C. Transduction was performed by adding the viruses to U251MG WT (wild-type) cell cultures in 6-well plates (Sarstedt; Numbrecht, Germany). After 24 h, the medium was replaced by a complete medium added with puromycin (1.5 µg/mL), performing cell selection for 5 days. After passage, the cells were individualized, forming clones. After 8 days, several clones were collected and transferred to 96-well plates (Nunc™; ThermoFisher Scientific) for amplification. After reaching confluency, cells were transferred to larger plates and cultured until freezing. To validate the U251MG Polκ mutated cells, two clones (B11 and B12) were selected for Sanger sequencing, western blotting, and in silico analyses. The B11 clone shows as compound-heterozygous (allele 1: 2-bp deletion with stop codon; allele 2: in-frame 12-bp/4-aa deletion), and the B12 clone as homozygous (both insertions and deletions occurred at the targeted genomic location that resulted in a net insertion of 2 bp in both alleles). Western blotting confirms the absence of TLS Polκ protein in the B12 clone and the presence of protein in the B11 clone once mutations are observed to promote truncated TLS Polκ protein. Experiments using cell viability (XTT assay) and cell death (Propidium Iodide staining) assay with TMZ confirm the phenotype similarly to B11 and B12 clones as knockout (KO).

XTT Assay

Cell Proliferation Kit II reagent (XTT; Cat. Nº 11465015001; Roche; Penzberg, Germany) was used in cell viability experiments. The principle of the technique is based on the colorimetric measurement of the reduction of tetrazolium salt into soluble formazan salt, and this reaction is dependent on cellular mitochondrial activity. Briefly, U251MG cells ($3x10^4$) were seeded in 35 mm plates; 72 h after the treatment, the culture medium was replaced by 500 µL of XTT mix diluted in PBS, following the manufacturer's specifications (Roche). The cells were incubated at 37ºC to metabolize the tetrazolium for approximately 1 h, and the formazan salt concentration was measured in a GloMax®-Multi Detection System (Promega, Madison, WI, USA) spectrophotometer at 490 nm and 750 nm. The ratio of absorbance values was used to calculate the percentage of cell survival using non-treated control cells as a reference.

Western Blotting

Total protein was extracted with lysis buffer (50 mM Tris–HCl pH 7.5, 20 mM NaCl, 1 mM MgCl2, 0.1% SDS, 1× protease inhibitor, 1× phosSTOP) and benzonase (250 U/mL: Cat. Nº 71206; Novagen, Merck) at for 20 min on ice. According to the manufacturer's instructions, total protein concentration was measured using a Pierce BCA protein assay kit (Cat. Nº 23227; ThermoFisher Scientific). Then, protein samples (40 µg) were treated with Bolt 4X LDS Sample Buffer (Cat. Nº B0007) and 10X Bolt™ Sample Reducing Agent (Cat. Nº B0009; ThermoFisher Scientific) and denatured at 70 °C for 10 min. The proteins were separated using the NuPAGE™ 4 to 12%, Bis-Tris (1.0 mm, 10 wells) Protein Gels (Cat. Nº NP0321BOX) and running at MiniGel Tank system (Cat. Nº A25977) with 1X NuPAGE MOPS SDS Running Buffer (Cat. Nº NP0001). After this, the proteins were transferred to PVDF membranes (iBlot™ 2 Transfer Stacks, PVDF, mini: Cat. Nº IB24002) using the iBlot2 Gel Transfer Device (Cat. Nº IB21001). The immunodetection was performed using primary antibodies against POLK (1:500 µL; Abcam, Cat. Nº ab90020; Rabbit) or GAPDH (1:500 µL; Abcam, Cat. Nº ab8245; Mouse) as the loading control. After, the membranes were incubated with Goat Anti-Rabbit IgG H&L conjugated and Goat Anti-Mouse IgG H&L conjugated with horseradish peroxidase (HRP; 1:5000 µL; Abcam; Cat. Nº ab205718; Cat. Nº ab205719) and revealed using the SuperSignal™ West Pico Substrate (Cat. Nº 34580). The iBright FL1500 imaging system (ThermoFisher Scientific) detected chemiluminescence signals.

Results

Legend Captions – Supplementary Files

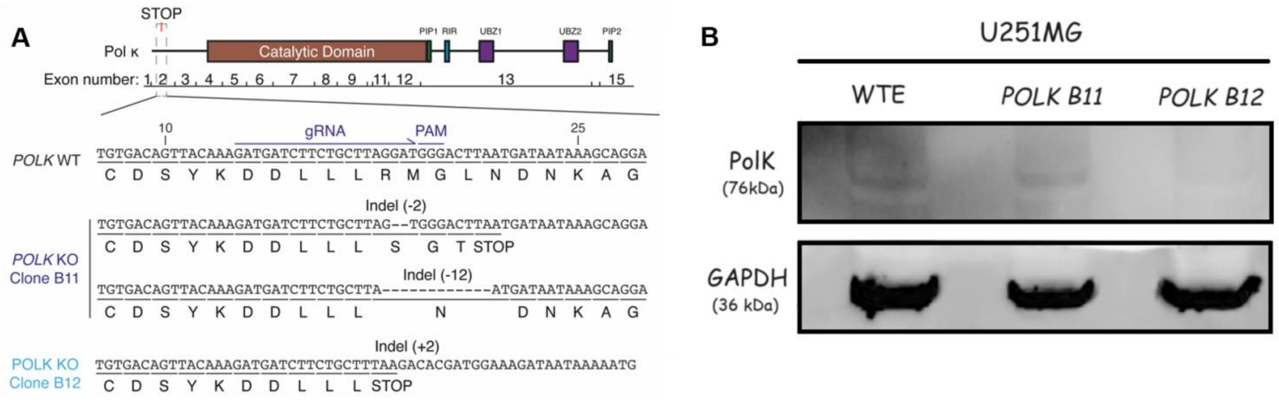

Figure S1. CRISPR/Cas9 Editing of the POLK Gene in U251MG Cell Clones. (A) Representation of CRISPR/Cas9 editing outcomes in U251MG cell clones targeting the POLK gene. The electropherogram depicts sequencing results of the wild-type (WT) U251MG cell line, with the guide RNA (underlined in solid blue), the PAM sequence (highlighted in purple), and the cleavage site (indicated by a vertical black dot). A comparison is shown between the WT sequence and the edited sequences of the B11 clone (compound heterozygous) and the B12 clone (homozygous) glioblastoma (GBM) cells with POLK knockout (KO). Sequencing data was adjusted for the reading phase and subjected to in silico analysis to predict amino acid sequences corresponding to each codon. The figure illustrates the wild-type reference sequence, the sequence of the B11 cell clone (with the arrow indicating possible heterozygosity), and the distinct alleles derived from the POLK KO cells originating from the B11 clone. Each allele exhibits a unique editing pattern, introducing premature stop codons in both cases. (B) Western blot analysis demonstrates the presence of Polκ protein in U251MG knockout cell clones following CRISPR/Cas9-mediated gene editing. The panels display protein bands corresponding to U251MG WT, Polκ KO B11 clone (compound heterozygous), and Polκ KO B12 clone (homozygous mutant). The top row indicates bands representing Polκ protein, while the bottom row shows bands for the normalizing protein GAPDH. The Western blot results reveal the formation of truncated Polκ protein in the B11 clone, indicative of successful editing and a functional Polκ protein fragment. Conversely, the B12 clone exhibits a complete absence of Polκ protein bands, confirming the successful generation of a homozygous mutant with a complete loss of Polκ protein expression.

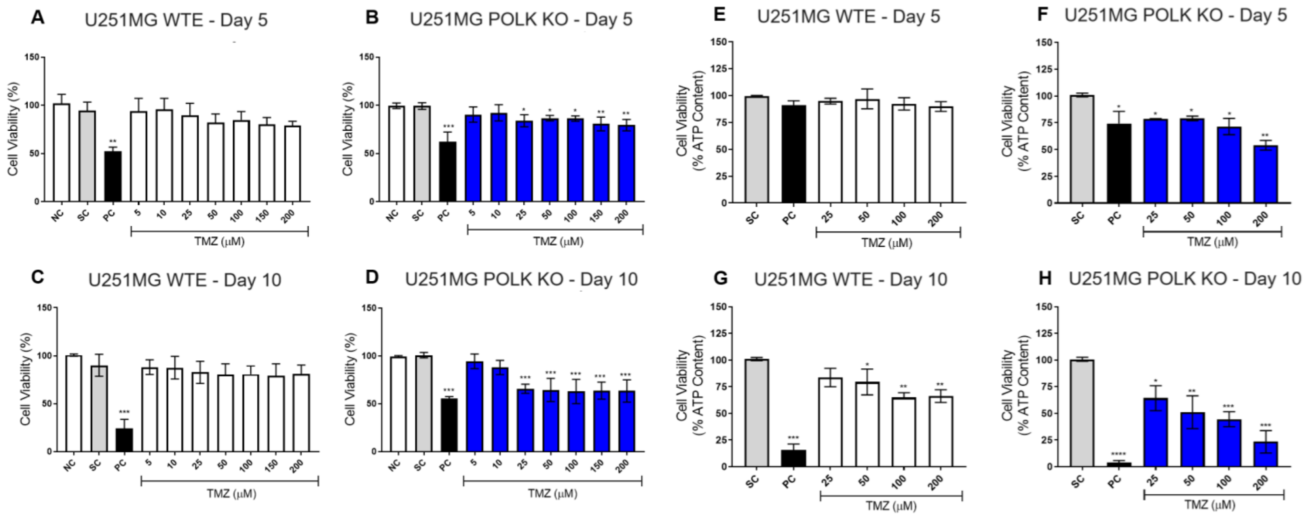

Figure S2. Cell viability assessment in GBM spheroids following TMZ treatment. (A-D) Cell viability (%) of U251MG WTE and TLS Polκ KO 3D tumor spheroids after exposure with TMZ and their respective controls at Day 5 (A/B) and Day 10 (C/D) as assessed by the resazurin assay. (E-H) Cell viability (% ATP Content) of U251MG WTE and TLS Polκ KO tumor spheroids after treatments with TMZ and respective controls at Day 5 (E/F) and Day 10 (G/H) as evaluated by CellTiter-Glo 3D ATP cell viability assay. All data were represented as means ± standard deviation (X±SD) from four spheroids (n = 4) per replicate to resazurin and three spheroids (n = 3) per replicate to CellTiter-Glo ATP and three independent biological experiments (n = 3). *Values statistically different from the SC group at the point (date) (*p < 0.05; **p < 0.01; ***p < 0.001; One-Way ANOVA followed by Dunnett post-test). NC: Negative control (DMEM HG); SC: Solvent Control (1% DMSO); PC: Positive Control (Cisplatin 100 μM); TMZ: Temozolomide.

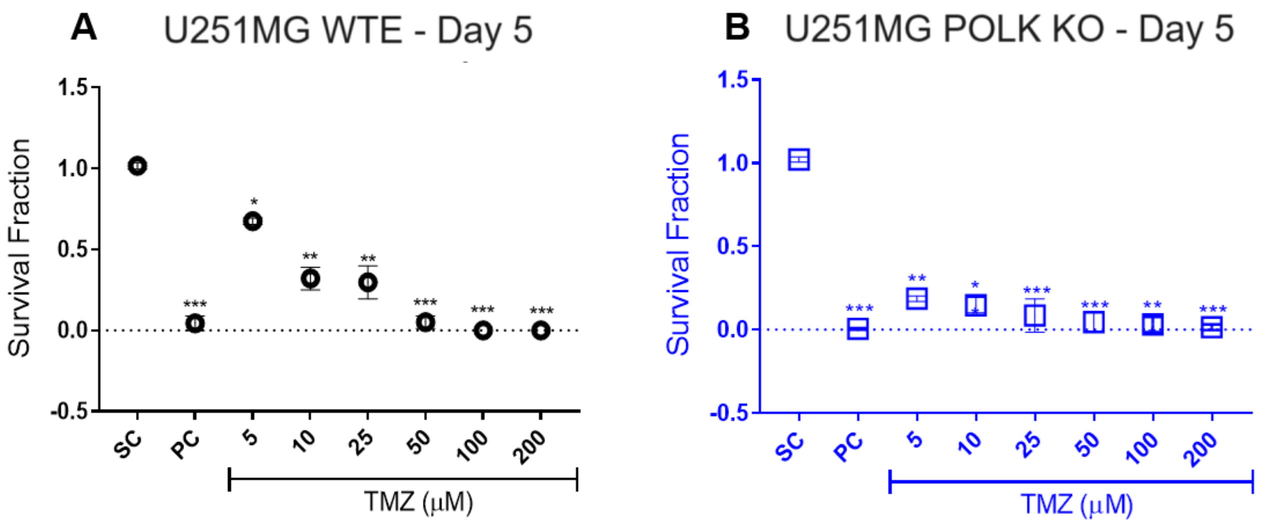

Figure S3. Cell survival in GBM spheroids after TMZ treatment. Survival fraction (SF) of U251MG WTE (A) and TLS Polκ KO (B) cells disaggregated from 3D tumor spheroids treated with TMZ and respective controls until Day 5. In the assay, colonies formed after 10 days of 3000 cells disaggregated from 3D tumor spheroids were analyzed. All data were represented as means ± standard deviation (X±SD) from six spheroids (n = 6) per well and three independent biological experiments (n = 3). *Values statistically different from the SC group cells at the point (date) (*$p < 0.05$; **$p < 0.01$; ***$p < 0.001$; ****$p < 0.0001$; One-way ANOVA followed by Dunnett post-test). SC: Solvent Control (1% DMSO); PC: Positive Control (Cisplatin 100 μM); TMZ: Temozolomide.

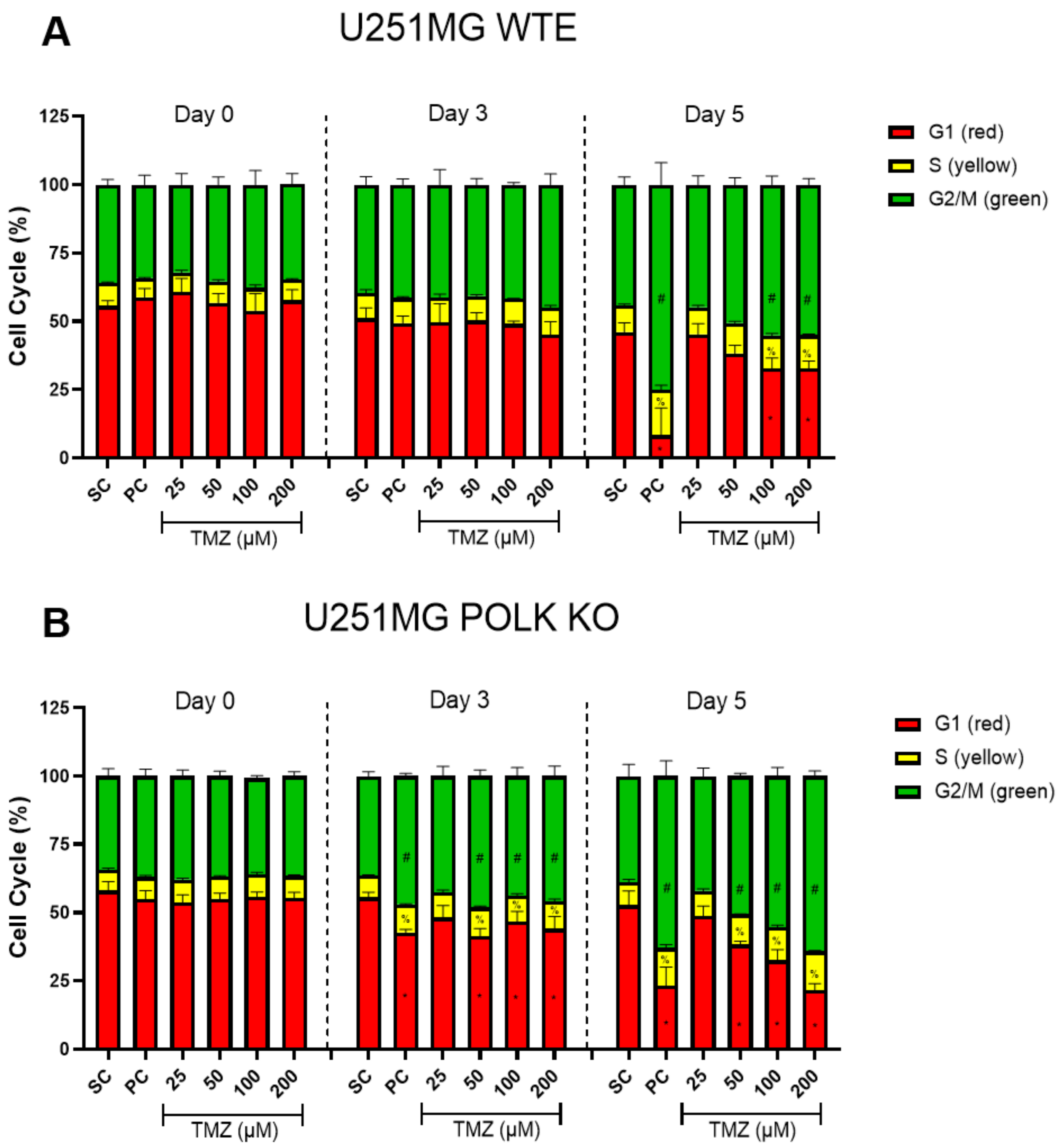

Figure S4. Analysis of cell cycle in GBM spheroids following TMZ treatment. (A-B) Cell Population (%) in each cell cycle phase in U251MG WTE (A) and TLS Polκ KO (B) 3D tumor spheroids expressing pBOB-EF1-FastFUCCI-Puro plasmid after Day 0, Day 3, and Day 5 of treatments with TMZ and their respective controls. All results were quantified from images at RFP (red) and FITC (green) channels using a 10x objective. The acquired images were analyzed and quantified at each channel using Fiji v. 3.1. Scale: 400 μm (white bar). All data were represented as means ± standard deviation (X±SD) from four spheroids (n = 4) per replicate and three independent biological experiments (n = 3). *Values statistically different from the WTE cells at the point (date) (*p < 0.05; **p < 0.01; ***p < 0.001; Two-way ANOVA followed by Bonferroni post-test). SC: Solvent Control (1% DMSO); PC: Positive Control (Cisplatin 100 μM); TMZ: Temozolomide.

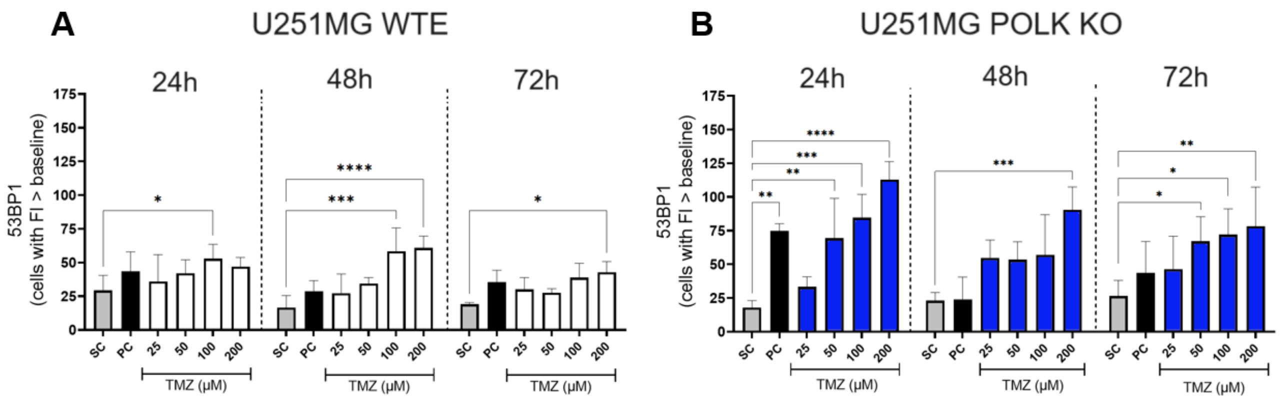

Figure S5. Evaluation of genotoxicity in GBM spheroids following TMZ treatment. (A-B) Cells with 53BP1-Apple-trunc fluorescence above the baseline in U251MG WTE (A) and TLS Polκ KO (B) 3D tumor spheroids were evaluated after 24-, 48- and 72-h treatments with TMZ and their respective controls. All data were represented as means ± standard deviation (X±SD) from four spheroids (n = 4) per replicate and three independent biological experiments (n = 3). *Values statistically different from the SC group at the point (date) (*p < 0.05; **p < 0.01; ***p < 0.001; ***p < 0.0001; ANOVA followed by Dunnett's post-test). SC: Solvent Control (1% DMSO); PC: Positive Control (Cisplatin 100 µM); TMZ: Temozolomide.

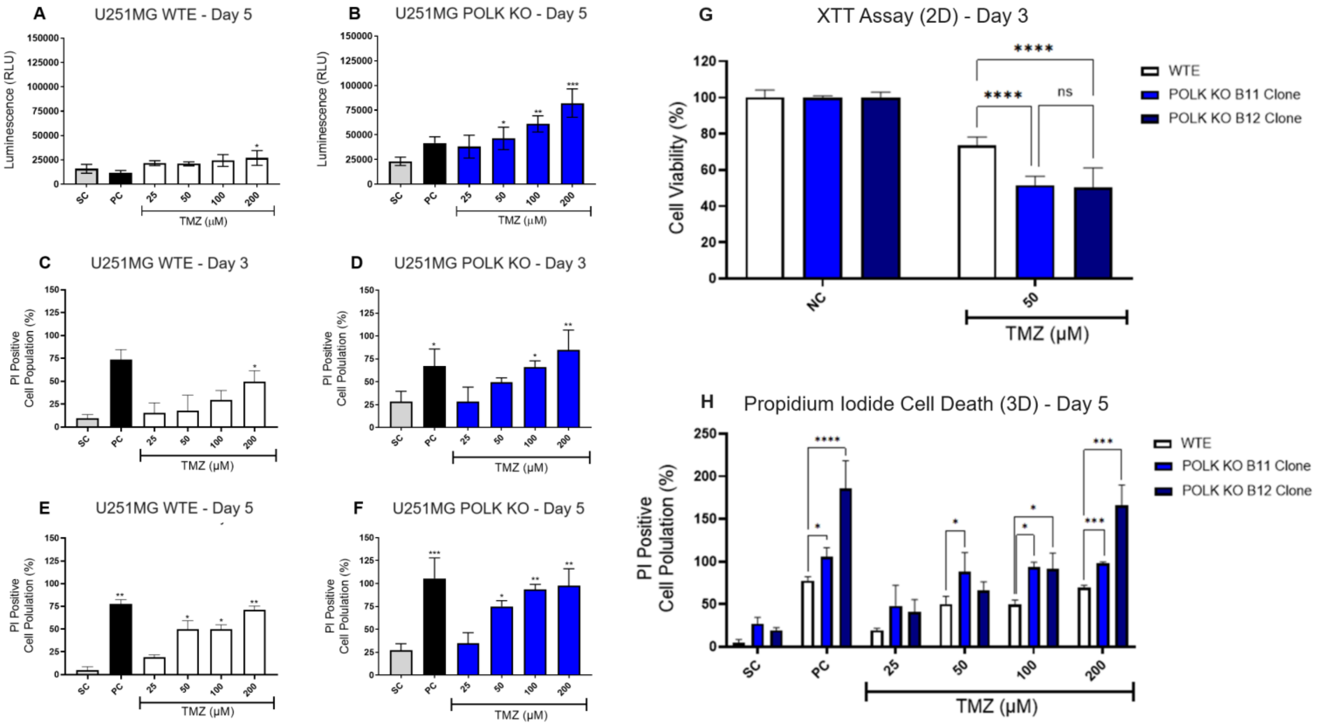

Figure S6. Assessment of cell death parameters in GBM spheroids following TMZ treatment. (A-B) Relative Luminescence (RLU) of Caspase 3/7 activity U251MG WTE (A) and TLS Polκ KO (B) 3D tumor spheroids after treatments with TMZ and respective controls at Day 5. (C-F) Cell Population (%) with Positive Propidium Iodide (PI) staining fluorescence in 3D tumor spheroids from U251MG WTE and TLS Polκ KO cells after treatments with TMZ and respective controls at Day 3 (C/D) and Day 5 (E/F). All data were represented as means ± standard deviation (X±SD) from four spheroids (n = 4) per replicate and three independent biological experiments (n = 3). *Values statistically different from the SC group at the point (date) (*p < 0.05; **p < 0.01; ***p < 0.001; ***p < 0.0001; ANOVA followed by Dunnett's post-test). (G) Cell viability (%) in TLS Polκ KO mutated cells (B11 and B12 clones) compared to U251MG WT cells following treatment with TMZ for 72 hours in 2D monolayer culture after performing XTT assay. All data were represented as means ± standard deviation (X±SD) from four technical replicates (n = 4) and three independent biological experiments (n = 3). (H) PI positive cell population (%) staining in 3D tumor spheroids derived from U251MG WT and TLS Polκ KO mutated cells (B11 and B12 clones) after treatment with TMZ and respective controls at Day 5. All data were represented as means ± standard deviation (X±SD) from five spheroids (n = 5). The data reveals comparable phenotypic sensitivity between the B11 clone (expressing truncated protein due to compound heterozygosity) and the B12 clone (a homozygous mutant with no protein expression) in 2D monolayer cultures. Furthermore, both B11 and B12 clones exhibit increased sensitivity to lower concentrations of TMZ in 3D spheroids. These findings suggest that CRISPR-mediated editing of the POLK gene renders U251MG GBM cells more sensitive to TMZ treatment, with both compound heterozygous and homozygous mutant clones exhibiting heightened sensitivity in 2D monolayers and 3D spheroids. (***p < 0.001 and **** p < 0.0001). NC: Negative Control (DMEM HG); SC: Solvent Control (1% DMSO); PC: Positive Control (Cisplatin 100 μM); TMZ: Temozolomide.

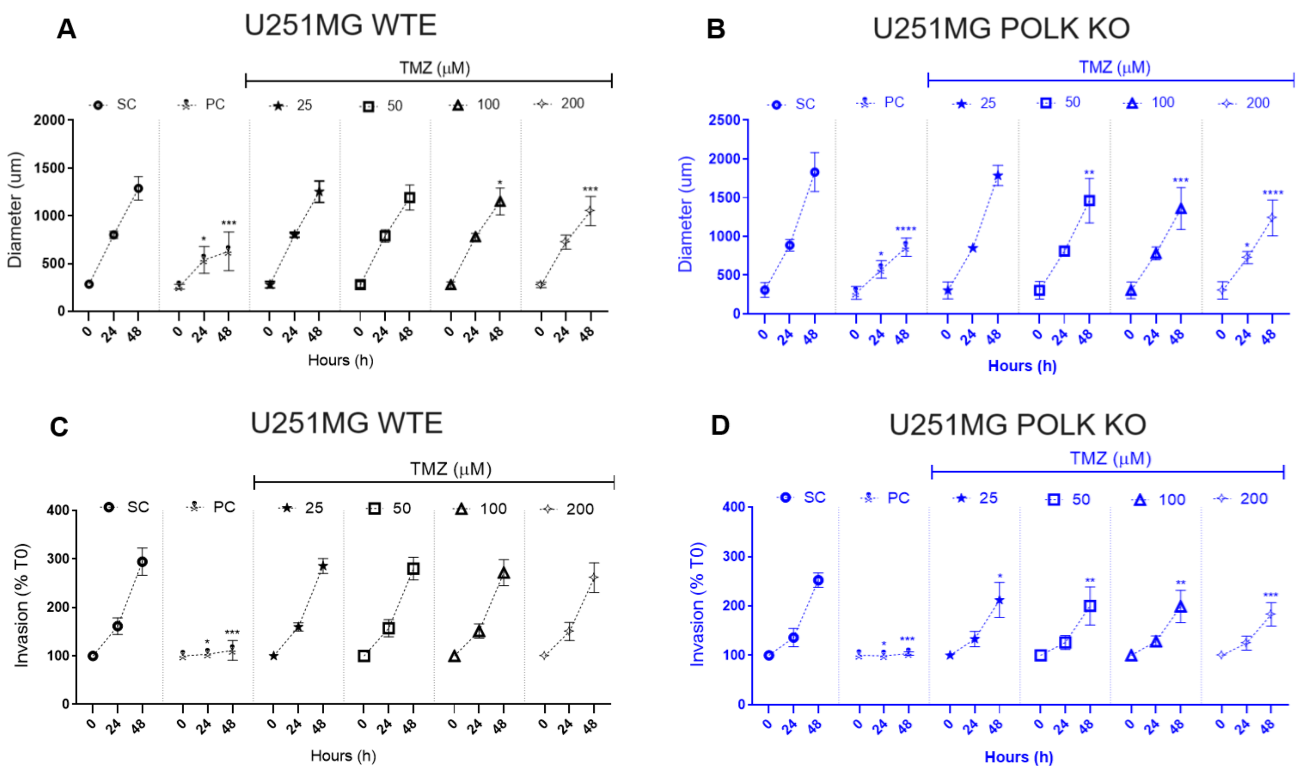

Figure S7. Quantifying metastatic parameters in GBM spheroids following TMZ treatment. (A-B) Diameter increase (µm) covered by U251MG WTE (A) and TLS Polκ KO cells (B) 3D tumor spheroids after treatments with TMZ and respective controls for 0-, 24-, and 48 h. The diameter (µm) of migration of the 3D tumor spheroids to the ECM after 0-, 24-, and 48 h were converted into % to the increase in diameter obtained at 0 h. (C-D) Percentage (%) of cell invasion (invadopodia) formed by U251MG WTE (C) and TLS Polκ KO cells (D) 3D tumor spheroids after treatments with TMZ and its respective controls for 0-, 24- and 48 h. The invasion area (μm^3) into the ECM (Matrigel) was converted to % considering the diameter increase of 0 h. Diameter analyses were performed using Zen 3.1 Blue Edition software (Carl Zeiss). All data were represented as means ± standard deviation (X±SD) from four spheroids (n = 4) per replicate and three independent biological experiments (n = 3). *Values statistically different from the SC group at the point (date) (*$p < 0.05$; **$p < 0.01$; ***$p < 0.001$; ***$p < 0.0001$; ANOVA followed by Dunnett's post-test). SC: Solvent Control (1% DMSO); PC: Positive Control (Cisplatin 100 µM); TMZ: Temozolomide.

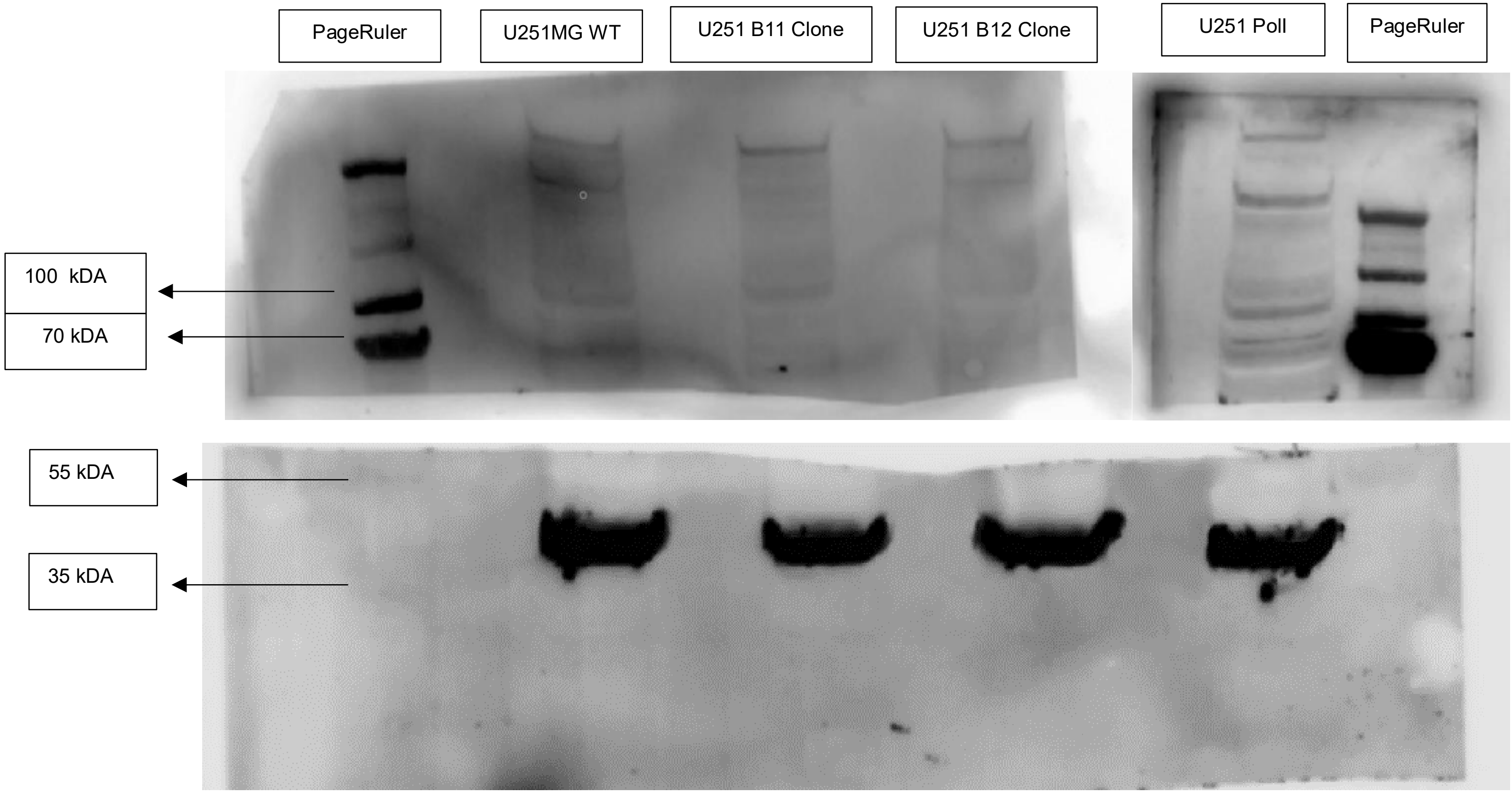

Western Blotting membrane was meticulously utilized to dissect and analyze protein expression patterns. The upper segment of the membrane was sectioned to accommodate the incubation of wells containing total proteins extracted from U251MG WT, PolK KO B11 Clone, and PolK KO B12 Clone cells with the primary antibody targeting POLK. Notably, a distinct piece corresponding to U251MG PolI KO cells was segregated for further analysis. Conversely, in the middle/lower region, the membrane facilitated the simultaneous assessment of GAPDH expression across four sequential samples of total proteins derived from U251MG WT, PolK KO B11 Clone, PolK KO B12 Clone, and PolI KO cells. This investigation involved incubation with the primary antibody specific to GAPDH, a protein characterized by its relatively low molecular weight. The PageRuler Plus Prestained Protein Ladder (ranging from 10 - 250 kDa) was a reliable marker for accurate molecular weight determination throughout experimentation. Notably, the anticipated molecular weights for DNA Polymerase Kappa/POLK antibody and GAPDH antibody were approximately 99 kDa and 36-40 kDa, respectively, providing critical reference points for precise interpretation of the results.
